# Interface engineering of cellobiose dehydrogenase improves interdomain electron transfer

**DOI:** 10.1002/pro.4702

**Published:** 2023-08-01

**Authors:** Thomas M. B. Reichhart, Stefan Scheiblbrandner, Christoph Sygmund, Wolfgang Harreither, Josef Schenkenfelder, Christopher Schulz, Alfons K. G. Felice, Lo Gorton, Roland Ludwig

**Affiliations:** ^1^ Biocatalysis and Biosensing Laboratory, Institute of Food Technology, Department of Food Science and Technology University of Natural Resources and Life Sciences (BOKU) Vienna Austria; ^2^ DirectSens GmbH Klosterneuburg Austria; ^3^ Department of Analytical Chemistry/Biochemistry Lund University Lund Sweden

**Keywords:** cytochrome, electron transfer, enzyme engineering, protein interface, surface charge

## Abstract

Cellobiose dehydrogenase (CDH) is a bioelectrocatalyst that enables direct electron transfer (DET) in biosensors and biofuel cells. The application of this bidomain hemoflavoenzyme for physiological glucose measurements is limited by its acidic pH optimum and slow interdomain electron transfer (IET) at pH 7.5. The reason for this rate‐limiting electron transfer step is electrostatic repulsion at the interface between the catalytic dehydrogenase domain and the electron mediating cytochrome domain (CYT). We applied rational interface engineering to accelerate the IET for the pH prevailing in blood or interstitial fluid. Phylogenetic and structural analyses guided the design of 17 variants in which acidic amino acids were mutated at the CYT domain. Five mutations (G71K, D160K, Q174K, D177K, M180K) increased the pH optimum and IET rate. Structure‐based analysis of the variants suggested two mechanisms explaining the improvements: electrostatic steering and stabilization of the closed state by hydrogen bonding. Combining the mutations into six combinatorial variants with up to five mutations shifted the pH optimum from 4.5 to 7.0 and increased the IET at pH 7.5 over 12‐fold from 0.1 to 1.24 s^−1^. While the mutants sustained a high enzymatic activity and even surpassed the IET of the wild‐type enzyme, the accumulated positive charges on the CYT domain decreased DET, highlighting the importance of CYT for IET and DET. This study shows that interface engineering is an effective strategy to shift the pH optimum and improve the IET of CDH, but future work needs to maintain the DET of the CYT domain for bioelectronic applications.

## Abstract 2

PDB Code(s): 4QI6;

**Abbreviations**
CDH: cellobiose dehydrogenase
CGM: continuous glucose monitoring
*Ch*CDH: CDH from *Crassicarpon hotsonii* (formerly *Myriococcum thermophilum*)
cyt *c*
: cytochrome *c*
CYT: cytochrome domain
DET: direct electron transfer
DH: dehydrogenase domain
IET: interdomain electron transfer
IR: interface region
MET: mediated electron transfer
MSA: multiple sequence alignment
WT: wild type

## 1 INTRODUCTION

Continuous glucose monitoring (CGM) sensors promise to improve diabetes management by providing an accurate and painless online measurement of the glucose concentration in blood or interstitial fluid (Didyuk et al., 2020). CGM sensors use enzyme‐based electrochemical sensors with an immobilized oxidoreductase as a biorecognition element and bioelectrocatalyst. Glucose biosensors have evolved from detecting either the decrease in concentration of the cosubstrate (O_2_) or the formation of the byproduct (H_2_O_2_) in glucose oxidase‐based first‐generation biosensors into second‐generation biosensors by employing electron mediators or redox polymers to facilitate the transfer of electrons from glucose oxidase (turnover number 130 s^−1^ at pH 7.4 and 25°C) (Kovačević et al., 2014) or glucose dehydrogenase (turnover number 418 s^−1^ at pH 7.4 and 30°C) (Sygmund et al., 2011) to the electrode via mediated electron transfer (MET). Typically, the efficiency of MET is the rate‐limiting step and governs the current output.

Third‐generation biosensors take advantage of oxidoreductases capable of direct‐electron transfer (DET) between the enzyme and the electrode (Milton & Minteer, 2017; Smutok et al., 2022). DET rates up to 580 s^−1^ have been observed on enzyme‐modified electrodes using flavoenzymes carrying a cytochrome domain (Ma et al., 2019). Ideally, third‐generation enzymes are not affected by redox‐active species and non‐susceptible to oxygen, thus avoiding interferences by many physiological or pharmaceutical electroactive substances and resolving the problem of fluctuating oxygen concentrations in blood (Jayakumar et al., 2022). These characteristics offer improved selectivity and sensitivity, a longer operational lifespan, and increased safety of the CGM sensor. Despite their clinical and commercial potential there is currently no implantable third‐generation CGM sensor available, partly because of the unmet requirements of a specialized glucose oxidoreductase with a high substrate specificity, catalytic activity, and DET, operating at the physiological pH of body fluids (Geiss et al., 2021).

The fungal hemoflavoenzyme cellobiose dehydrogenase (CDH, EC 1.1.99.18, CAZy family AA3_1) is a promising third‐generation enzyme that has been tested as biorecognition element in carbohydrate detecting biosensors an as bioelectrocatalyst in biofuel cells and biosupercapacitors (Scheiblbrandner et al., 2022). CDH is secreted by wood‐degrading fungi to assist lytic polysaccharide monooxygenase as an auxiliary enzyme in the oxidative depolymerization of polysaccharides at acidic pH (Kracher et al., 2016). Its bidomain structure consists of a mobile heme *b*‐containing cytochrome domain (CYT) which is tethered to a catalytic FAD‐binding dehydrogenase domain (DH) by a flexible linker (Felice et al., 2021; Tan et al., 2015). CDH transfers electrons to the electrode in three steps: (i) substrate oxidation and reduction of the FAD in the DH domain, (ii) interdomain electron transfer (IET) from FADH_2_ to the heme *b* in the CYT domain, and (iii) DET from the reduced heme to the terminal electron acceptor. The natural electron acceptor lytic polysaccharide monooxygenase is contacted by the heme of CYT and cytochrome *c* is supposed to work similarly (Ma et al., 2019). During this process, CDH changes between two conformations: the closed state and the open state (Bodenheimer et al., 2018). In the IET‐competent closed state, the CYT domain is in contact with the DH domain sharing a small interface and an edge‐to‐edge distance between the cofactors of ~8 Å (Kracher et al., 2016). In the DET‐competent open state, the CYT and DH domains are separated with a maximum distance of up to 50 Å between the cofactors (Tan et al., 2015), which allows the CYT domain to contact macromolecular electron acceptors such as an electrode surface (Ma et al., 2019). This interdomain flip‐flop motion between the CYT and DH domain is a dynamic process that regulates and governs the electron transfer process (Harada et al., 2017), which is faster at acidic pH, but slows down considerably at neutral pH (Harreither et al., 2012; Igarashi et al., 2002). Structural studies using small‐angle neutron scattering and small‐angle x‐ray scattering support this mechanism and demonstrate that dynamics of the electron transfer process depend on the pH and presence of divalent cations (Bodenheimer et al., 2017).

The ability of CDH to perform DET has many potential applications in bioelectronics (Kracher et al., 2016). CDH enables third‐generation sensor architectures that can operate at a low working potential, thus omitting electrochemical interferences commonly observed with other glucose biosensors (Felice et al., 2013; Halbmayr‐Jech et al., 2020). CDH‐based biosensors were first realized on gold (Lindgren et al., 2001) and carbon (Harreither et al., 2007) electrodes and later on nanostructured electrodes based on carbon nanotubes (Tasca et al., 2011) or gold particles (Wang et al., 2012), showing improved current output. These advanced electrode materials enable the construction of biosensors and biofuel cells operating in complex physiological fluids such as sweat (Falk et al., 2014) and tears (Falk et al., 2012). Since CDH variants with a sufficiently high substrate specificity for glucose exist in nature (Harreither et al., 2011) and have been engineered for improved glucose specificity and activity (Ortiz et al., 2017), CDH can be designed for biomedical applications such as online glucose monitoring for diabetes management (Felice et al., 2013).

Despite these promising features, CDH faces a significant shortcoming that hampers its application in CGM sensor: the acidic pH optimum of most CDHs exhibits only marginal IET at neutral pH, like for the well‐characterized CDH from *Crassicarpon hotsonii* (formerly *Myriococcum thermophilum*, *Ch*CDH) (Kracher et al., 2016). Stopped‐flow experiments revealed that the IET between DH and CYT, while always rate‐limiting, shuts down above pH 6, despite the still very efficient glucose conversion inside the DH domain at neutral pH (Igarashi et al., 2002; Kadek et al., 2017). The measured currents of CDH‐based biosensors operating at physiological pH are therefore severely limited by the IET rate (Harreither et al., 2012; Meneghello et al., 2019). Studies suggest that the IET at neutral pH is disrupted by electrostatic repulsion between deprotonated Asp and Glu side chains at the CYT/DH interface, thus preventing the formation of the IET‐competent closed state of CDH (Kadek et al., 2017, 2015; Kracher et al., 2015). One strategy to overcome this electrostatic repulsion is to exert the bridging effect of divalent metal cations such as Ca^2+^ (Schulz et al., 2012). This IET‐enhancing effect of Ca^2+^ has been shown to enhance *Ch*CDH's turnover rate in solution and catalytic currents on electrodes by up to 200‐ and 5‐fold, respectively (Schulz et al., 2012). However, while this approach is helpful for single‐use, disposable biosensor systems, it is not suitable for CGM sensors.

To engineer *Ch*CDH into an efficient biocatalyst with an efficient IET rate at neutral pH, we employed interface engineering of the CYT domain to change unfavorable into favorable electrostatic interactions at the CYT/DH domain interface. The interface engineering approach to improve the IET of CDH at physiological pH is based on the hypothesis that electrostatic repulsion between CDH's interface at neutral pH can be overcome by either replacing acidic side chains by neutral, polar side chains or introducing alkaline side chains on specific interface regions (IR). As a model enzyme, we chose *Crassicarpon hotsonii* CDH, which is one of the best studied CDHs to date (Kracher et al., 2016) and for which a high‐resolution crystal structure in the closed state is available (Tan et al., 2015). To not interfere with the catalytic properties of the DH domain, we focused on the engineering on the CYT domain. First, we used structural and phylogenetic analyses to select suitable engineering positions on the CYT domain. Next, we performed a proof‐of‐concept study on CYT domain residue D160, proving the feasibility of the interface engineering strategy. Based on these findings, 17 single‐site variants were engineered, produced, and characterized, which resulted in 5 variants with enhanced IET at neutral pH. Then, we combined these promising mutations into six combinatorial variants to yield synergistic effects. Finally, three promising variants immobilized onto electrodes were electrochemically characterized via cyclic voltammetry and amperometry in flow injection analysis mode.

## 2 RESULTS

### 2.1 Surface electrostatic models revealed regions and residues for interface engineering

**FIGURE 1:**
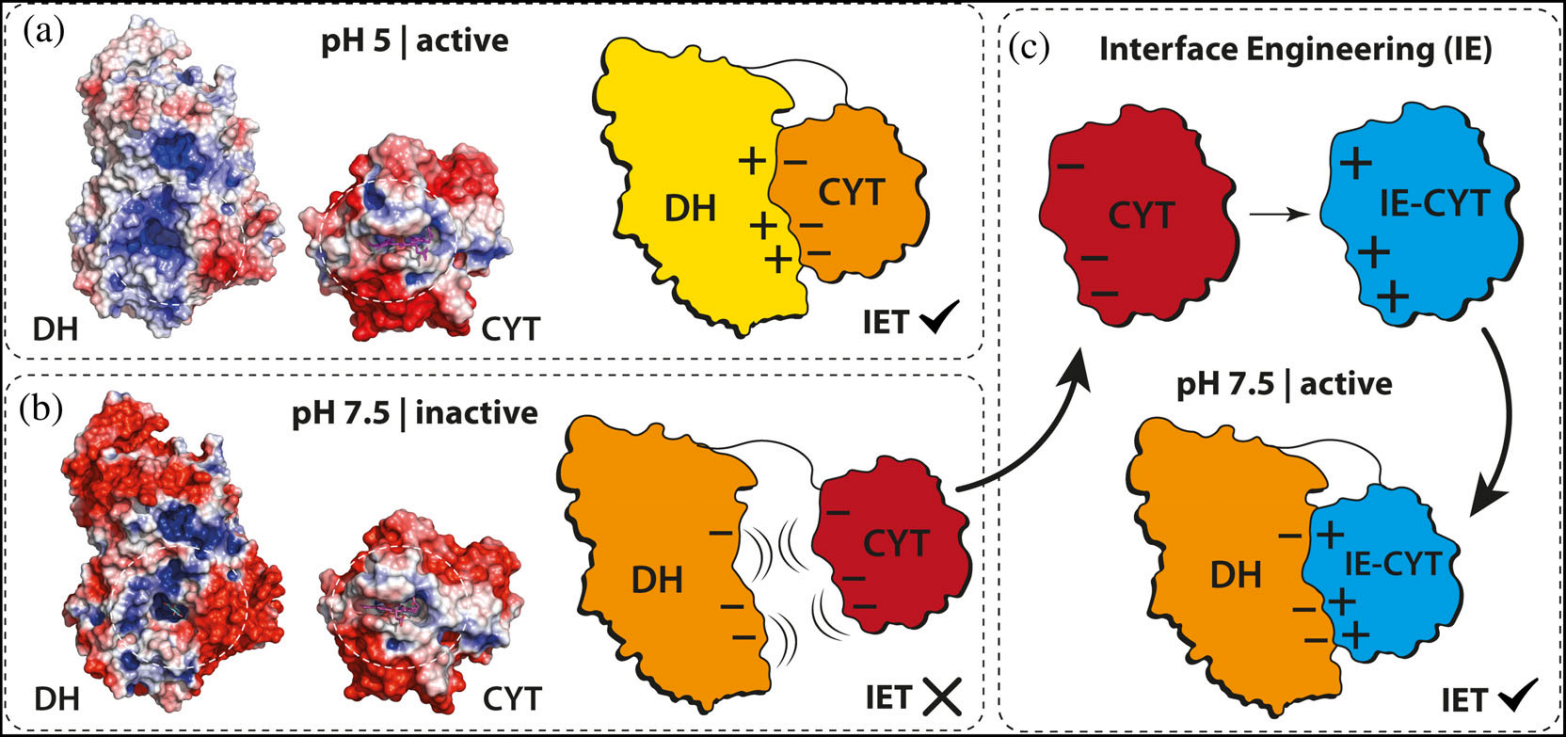
Surface electrostatics and interface engineering of *Ch*CDH. (a, b) Protein surface electrostatics and schematics of DH/CYT domain interactions at pH 5.0 (a) and pH 7.5 (b) are shown for the DH and CYT domain of *Ch*CDH. The dashed circles on the DH and CYT domain roughly indicate the DH/CYT interaction site derived from the crystal structure in the closed state (PDB: 4qi6). Surface electrostatic profiles were calculated using the Adaptive Poisson–Boltzmann Solver (APBS) and are shown as a color gradient from red to blue (−2 to +2 k_B_T/e). Of note, the cyt *c* cofactor (shown as sticks) was excluded from APBS calculations. At pH 5.0, the DH domain (yellow) and CYT domain (orange) have complementary surface charges at the interface and are thus capable of IET. At pH 7.5, surface charge alterations of the DH (orange) and CYT domain (red) cause repulsive electrostatics, annihilating IET. (c) Interface engineering strategy to overcome charge incompatibility at pH 7.5. *Top*: Acidic amino acids at the negatively charged CYT domain (red) are replaced with alkaline residues (mostly lysine) to engineer a positively charged CYT domain (blue) at pH 7.5. *Bottom*: IE‐CDH with a positively charged CYT can achieve the active and IET‐competent closed state.

To identify suitable interface engineering targets on the CYT domain, we first analyzed the pH‐dependent protein surface electrostatics at the CYT/DH interface (Figure 1). At acidic conditions, the interface of the DH domain is mostly positively charged while the interface of the CYT domain is slightly negatively charged, which results in complementary, attractive surface electrostatics of both domains (Figure 1a). At neutral pH conditions which is relevant for blood glucose measurements, both domains become predominantly negatively charged and the resulting electrostatic repulsion hinders the formation of the closed, IET‐competent state (Figure 1b). In particular, the negatively charged E304–D307 loop harbors highly conserved aspartate and glutamate residues that may contribute to electrostatic repulsion (Figure S1, central left panel). The effect of electrostatic repulsion is also evident in a drop of the IET rate to 9% at pH 7.5 relative to the pH optimum of *Ch*CDH‐WT (Table 1).

**TABLE 1:** pH optima and IET rates of single‐site variants.^a^

		pH optimum		pH 4.5		pH 7.5	
Interface region	Variant	pH optimum	ΔpH versus WT	IET rate (s^−1^)	Fold‐change	IET rate (s^−1^)	Fold‐change
‐	WT	4.5	0	1.14 ± 0.08	1.0	0.10 ± 0.01	1.0
IR‐1	G71K	5.0	+0.5	0.67 ± 0.09	0.6	0.10 ± 0.01	1.0
	K72	5.5	+1.5	0.02 ± 0.01	0.0	na	na
	G72K	na	na	na	na	na	na
	N76K	4.0	−0.5	0.02 ± 0.01	0.0	na	na
	T97K	4.5	0	0.71 ± 0.08	0.6	0.07 ± 0.01	0.7
IR‐2	F159K	5.0	+0.5	0.02 ± 0.01	0.0	na	na
	F159R	5.0	+0.5	0.32 ± 0.01	0.3	0.03 ± 0.01	0.3
	D160G	5.0	+0.5	0.29 ± 0.07	0.3	0.03 ± 0.01	0.3
	D160K	5.0	+0.5	2.4 ± 0.01	2.1	0.68 ± 0.09	6.8
	D160R	5.0	+0.5	1.34 ± 0.04	1.2	0.30 ± 0.04	3.0
	Q174K	5.0	+0.5	0.76 ± 0.02	0.7	0.14 ± 0.01	1.4
	D177K	5.0	+0.5	0.93 ± 0.36	0.8	0.29 ± 0.04	2.9
IR‐3	W155K	5.0	+0.5	0.03 ± 0.20	0.0	na	na
	W155R	4.0	−1.0	0.04 ± 0.08	0.0	na	na
	Q157K	4.5	0	0.59 ± 0.19	0.5	0.06 ± 0.01	0.6
	M180K	5.0	+0.5	1.14 ± 0.11	1.0	0.78 ± 0.07	7.8
	I182K	na	na	na	na	na	na

Abbreviation: na, not active.

^a^ Values are arithmetic means ± 95% confidence intervals of three independent experiments.

Based on the surface electrostatic models, we hypothesized that the overall charge polarity at the CYT/DH interface could be altered through mutagenesis of specific residues in a way that attractive electrostatic interactions would favor formation of the IET‐competent closed complex at neutral pH. If this hypothesis is correct, then removing acidic residues (negatively charged at neutral pH) or introducing alkaline residues (positively charged at neutral pH) at the CYT domain interface could increase the IET at neutral pH by restoring the charge compatibility with the DH domain (Figure 1c).

### 2.2 Structural and phylogenetic analyses guided the selection of interface engineering targets

To select suitable targets for interface engineering, we used two complementary computational methods: structural inspection combined with phylogenetic analysis.

Structural analysis of the high‐resolution crystal structure of *Ch*CDH in the closed conformation (PDB: 4QI6) using the python script interfaceResidues.py (see Section 5) showed that 64 amino acids at the CYT and DH domain form the interface (Table S1, Figure S2). The interface residues consisted of 31 hydrophobic (48%), 25 polar (39%), and 8 ionizable amino acids (13%). On the CYT domain interface, only two ionizable amino acids (D160 and D177) can be found, which we both selected as engineering targets. In addition, we aimed to select suitable hydrophobic and polar amino acids for the insertion of alkaline amino acids on the CYT interface. To select these residues, we performed multiple sequence alignment (MSA) and phylogenetic analysis of 362 CDH sequences to investigate the sequence homology and conservation rate of interesting positions derived from the structural analysis (Figure S3).

**FIGURE 2:**
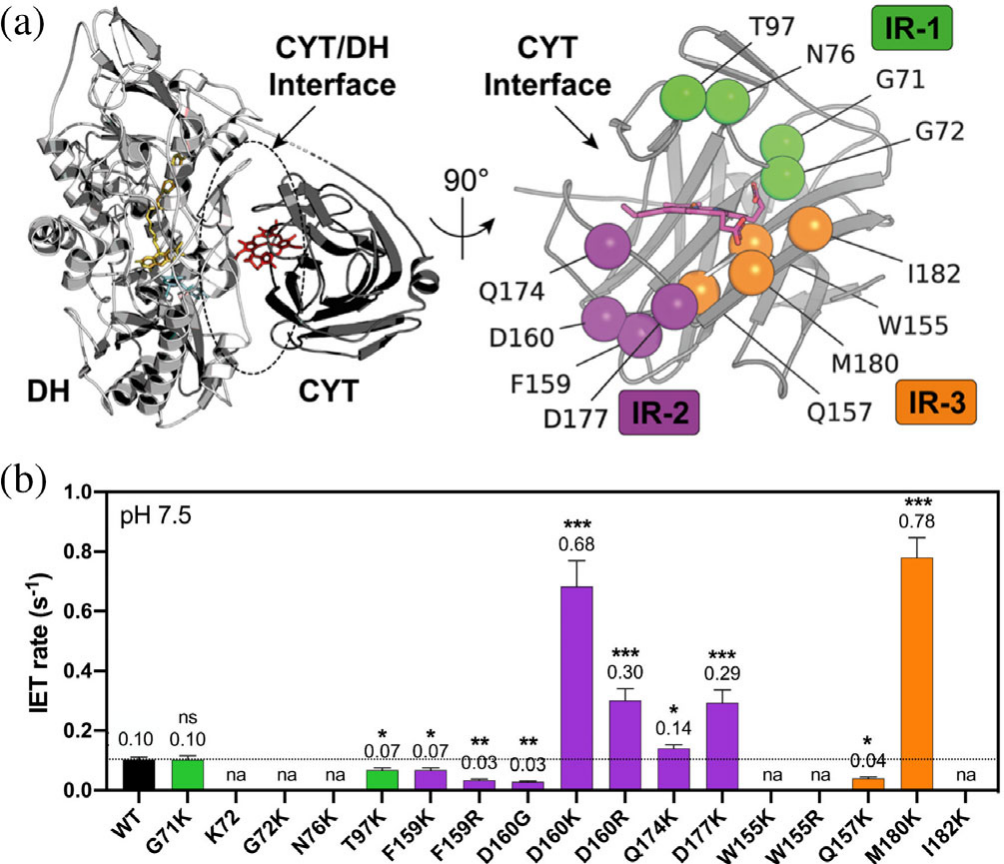
Interface engineering of the CYT domain shifts the pH optimum and enhances the IET rate at pH 7.5. (a) Molecular structure of *Ch*CDH (left) and the CYT domain interface (right) with engineering targets shown as spheres divided into three interface regions IR‐1 (green), IR‐2 (purple), and IR‐3 (orange). Cartoon representations were prepared in PyMOL using the crystal structure of *Ch*CDH (PDB: 4QI6). (b) IET rates at pH 7.5 of the variants measured with the cyt *c* assay. Values are arithmetic means ± 95% confidence intervals of three independent experiments. Asterisks indicate the statistical significance with *p* < 0.0001 (***), *p* < 0.001 (**), and *p* < 0.05 (*). The dotted lines indicate the *Ch*CDH‐WT values. For K72, a lysine residue was inserted between G71 and G72. Of note, the variants G17K, K72, N76K, F159K, W155K, W155R, I182K were inactive at pH 4.5 but not pH 7.5 (as defined by an IET rate of ≤0.01 s^−1^, see Figure S5). na, not active; ns, not significant.

By combining MSA and structural analysis, we identified 12 potential mutagenesis targets, which we grouped into the three interface regions: IR‐1, IR‐2, and IR‐3 (Figure 2a). On IR‐1, we selected G71, G72, N76, and T97, which are located on a beta‐hairpin at the core of the CYT interface and facing four acidic amino acids on the DH domain (E304, D305, D307, and D642). On IR‐2, we chose F159, D160, Q174, and D177—all facing D297 on the DH domain as the central acidic residue located at the interface core. On IR‐3, we picked W155, Q157, M180, and I182 due to their vicinity to E550, D553, and D554 on the DH domain. The reason for selecting these 12 mutagenesis targets was that their frequency distribution revealed a low conservation rate (i.e., high variability) and alternative polar and alkaline amino acids (Figure S3). For instance, residues G71, N76, W155, Q157, F159, and D160 showed alternative arginine and lysine residues, indicating that mutations toward alkaline residues are feasible.

Altogether, the structural and sequence analyses were useful to select 12 promising mutagenesis targets on three interface regions of the CYT domain. The presence of alternative polar and uncharged residues on the selected targets suggests that mutations at these positions are feasible and might improve charge compatibility at neutral pH.

### 2.3 Proof of concept on D160 verified the feasibility of our interface engineering strategy

After selecting suitable engineering targets, we performed a proof‐of‐concept study on D160 to test the effects of replacing an acidic CYT interface residue with another amino acid. Of the three available positively charged residues (arginine, histidine, and lysine), we chose arginine (D160R) and lysine (D160K) as the most promising side chains from the phylogenetic analysis (Figure S3). The side chains of arginine and lysine also have the advantage of being highly flexible and thus can orient themselves in the most suitable conformation in the interface. We also included an uncharged variant (D160G) to investigate the effects of removing an entire side chain and negative charge from the CYT interface.

To test the mutagenesis effects on D160, we recombinantly produced *Ch*CDH‐WT and the three variants in *K. phaffii* and purified the enzymes to homogeneity using hydrophobic interaction and anion exchange chromatography. The reduction of cyt *c* in the presence of substrate was used to spectrophotometrically measure and compare the IET rate of the purified variants. The cyt *c* assay measures the IET rate since cyt *c* is specifically reduced by the CYT domain, which can only occur after substrate oxidation in the DH domain and the rate‐limiting IET to the CYT domain. The pH profile of the recombinantly produced *Ch*CDH‐WT variant agrees with previously published data (Kracher et al., 2015; Zámocký et al., 2008), showing an IET pH optimum at pH 4.5. The pH profiles of the D160G, D160K, and D160R (Figure S4A) revealed that all three variants shifted the pH optimum of *Ch*CDH toward the neutral range compared to the WT (Table 1). While D160G showed only 28% of the WT IET rate at pH 7.5, D160R and D160K significantly increased the IET rate of CDH at pH 7.5 by 3.0‐ and 6.8‐fold, respectively (Figure S4B). Overall, these findings suggest that removing a negative charge can shift the IET pH optimum but a replacement with a positive charge is necessary to also enhance the IET rate at neutral pH.

This proof of concept supported our working hypothesis that replacing an acidic with an alkaline amino acid at the CYT domain interface can shift the pH optimum and increase the IET in the neutral pH range. The increased IET rate indicates an improved CYT/DH complex formation of *Ch*CDH resulting in a more frequent closed state conformation, which is IET competent. Based on the fact that D160K had a higher IET than D160R, we chose lysine for the site‐directed replacement of the remaining engineering targets. However, two arginine mutations (W155R and F159R) were also tested since they were suggested by the phylogenetic analysis (Figure S3).

### 2.4 Single‐site variants shift the pH optimum and improve electron transfer rates

Next, we engineered 17 single‐site variants of *Ch*CDH on the three selected interface regions IR‐1, IR‐2, and IR‐3 and characterized the pH‐dependent IET rate of the purified enzymes (Figures 2a and S1,S2). To judge whether a mutant was active, the threshold for a reliable measurement of the IET rates was set at 0.01 s^−1^ based on the limit of quantification determined for the cyt *c* assay (Figure S5). Of the 17 variants, 15 variants (88%) were active and showed an intact IET, 11 variants (73%) showed a neutrally shifted pH optimum by 0.5–1.5, and five variants (33%) had an increased IET rate at pH 7.5 with 1.4 to 7.8‐fold above the WT (Figure S6, Table 1). To better understand the causes and effects of the mutations, we analyzed changes in the pH optimum and IET rate at pH 7.5 on each interface region.

On IR‐1, four variants (G71K, K72, N76K, and T97K) were active at the pH optimum, of which two (G71K and K72) showed a neutrally shifted pH optimum, and two variants (G71K and T97K) showed IET at pH 7.5 similar to the WT (Figure 2b, Table 1). The loss of IET at pH 7.5 for K72, G72K, and N76K indicates that these mutations may disrupt the CYT/DH interface and impede the formation of the IET‐competent closed state. The most probable explanation for these findings is that the mutations disrupted cofactor binding or orientation due to their close proximity to M74, which coordinates the heme *b* cofactor together with H176. Regarding shifts in pH optimum, IR‐1 mutants showed mixed results. While G71K and K72 showed a slight shift of the pH optimum toward neutral pH, N76K showed an acidic shift and T97K had no effect. K72 with a lysine insertion between G71 and G72 showed the greatest pH shift from 4.5 to 6.0 but had only 3% of the WT IET rate at the pH optimum and was inactive at pH 7.5. Similarly, N76K showed only 3% of the WT IET rate at the pH optimum and was also inactive at pH 7.5. Overall, the mutagenesis studies indicate that IR‐1 is not a good target region to engineer the IET pH optimum of *Ch*CDH toward the neutral pH range. Only the mutant G71K showed a neutrally shifted pH optimum and an intact IET, making it a promising variant for further studies.

On IR‐2, all seven variants shifted the pH optimum toward the neutral range and four variants (D160K, D160R, Q174K, and D177K) improved the IET at pH 7.5 by 1.4–6.6‐fold (Figure 2b, Table 1). These findings demonstrate that IR‐2 is a potential hot spot to engineer and modulate the pH‐dependent IET rate of *Ch*CDH. An explanation for this finding is that positive charges at this interface region alleviate the electrostatic repulsion between the CYT and DH domain via long‐range electrostatic interactions (described below). Based on these findings, we selected IR‐2 mutants D160K, Q174K, and D177K for the construction of combinatorial variants.

On IR‐3, four of five variants (W155K, W155R, Q157K, and M180K) were active at pH 4.5 and two mutants (Q157K and M180K) showed an intact IET at pH 7.5. (Figure 2b, Table 1) Altogether, the rationally designed mutant M180K showed the highest IET rate at pH 7.5 of all variants tested, which was 7.8‐fold higher than the WT (Figure 2b, Table 1). This was unexpected since M180K was not supported by the phylogenetic analysis. Interestingly, and in contrast to the other variants, M180K improved IET only at pH 7.5 but not at pH 4.5. This becomes apparent when plotting the IET rates at pH 4.5 (the pH optimum of *Ch*CDH) against the IET rates at pH 7.5, showing M180K as an outlier (Figure S7). By performing a linear regression analysis, we found a significant correlation between the IET rates at pH 4.5 and 7.5 (*r* = 0.80, 95% CI 0.33 to 0.95, *p* = 0.0029). In other words, variants with enhanced IET rates at pH 7.5 also showed an increased IET rate at pH 4.5, indicating that the mutants improved the overall activity of *Ch*CDH. In contrast, M180K showed its improvements only at pH 7.5. Hence, when removing M180K from the correlation analysis, the Pearson correlation coefficient was increased even further (*r* = 0.98, 95% CI 0.89–0.99, *p* < 0.0001), indicating that M180K is qualitatively different from other variants. As such, we hypothesize that M180K may operate by a different mechanism than the other variants (as discussed below).

To summarize the single‐site mutagenesis study, we found significant improvements in the pH optimum and IET rate at pH 7.5 for five single‐site variants: G71K, D160K, Q174K, and M180 (Table 1). Interestingly, M180K showed the greatest improvements in IET at pH 7.5 but no changes in IET at pH 4.5 compared to the WT, which sets M180K apart from all the other variants. Overall, the high rate of active and improved variants indicates the effectiveness of our interface engineering strategy.

### 2.5 Putative mechanisms of electrostatic steering and heme *b* cofactor stabilization

**FIGURE 3:**
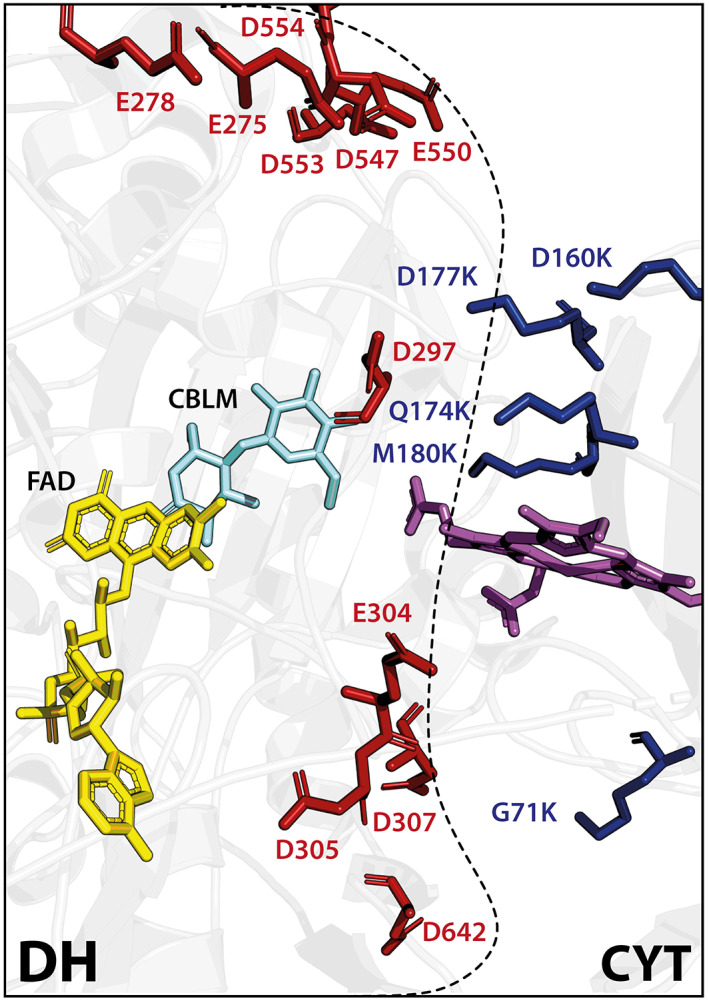
Putative mechanisms of electrostatic steering and heme *b* cofactor stabilization. CDH interface of the DH (left) and CYT domain (right) are shown. IE‐variants G71K, D160K, D177K, and M180K are shown as blue sticks. Sticks are also shown for the negatively charged DH domain interface residues E275, E278, E304, D305, D307, D547, E550, D553, and D642 (red), the FAD cofactor (yellow), the heme *b* cofactor (magenta), and the inactive substrate analog cellobiono‐δ‐lactame (CBLM, cyan). The cartoon model of *Ch*CDH (PDD: 4QI6) is shown in light gray. The dotted line indicates the interface.

Based on the single‐site mutagenesis study, we suggest two mechanisms to explain how the interface mutations shift the pH optimum and improve the IET rate of *Ch*CDH: (1) electrostatic steering via long‐range attractive charge interactions and (2) stabilization of the heme *b* cofactor inside the propionate docking site via hydrogen bonding of M180K (Figure 3).

The first mechanism, electrostatic steering, is based on the theory of long‐range electrostatic interactions, which are weakly specific forces conferred by surface charges shown to dominate the kinetics of protein–protein interactions (Sheinerman et al., 2000). In contrast to short‐range electrostatic forces such as salt bridges, which are strongly specific and typically occur at charge‐to‐charge distances of up to ~4 Å, long‐range electrostatic interactions occur at distances of ≥5 Å, typically within 5–13 Å (Maleki et al., 2013; Zhou & Pang, 2018). As such, we hypothesize that our variants improve the kinetics of CYT/DH complex formation and IET rates at pH 7.5 via electrostatic steering effects mediated by long‐range electrostatic charges. To test this hypothesis, we revisited our structural analysis and calculated charge‐to‐charge distances of the improved mutants on the CYT domain (G71K, D160K, Q174K, D166K, and M180K) with acidic residues on the DH domain (Figure 3, Table S2). To assess the likelihood of a mutated residue to engage in long‐range electrostatic interactions, we chose a cut‐off distance of 13 Å (Maleki et al., 2013; Zhou & Pang, 2018).

On IR‐1, G71K opposes D307 and D642, which are located at the outer rim of the interface, with a distance between 9 and 10 Å. On IR‐2, all variants face D297 as the central negative charge on the DH domain interface, but are also in proximity to K299, which may dampen the attractive long‐range electrostatic interactions with D297. However, the IR‐2 mutants may also interact with other residues than D297, given their vicinity to other negatively charged side‐chains. For instance, D160K may develop long‐range electrostatic attraction with E275 and D553; Q174K with E304; and D177K with E275, D547, E550, and D642 as well as the heme *b* propionate A moiety. The fact that D160K and D177K have more potential interaction partners than Q174K (3 and 6 compared to 2, respectively) can also explain why D160K and D177K exhibit higher IET rates than Q174K (Figure 2, Table 1).

Overall, the model of electrostatic steering can well explain the improvements seen in our mutants on IR‐1 and IR‐2. However, the electrostatic steering model cannot explain two effects observed for the IR‐3 variant M180K, which is unlikely to contribute to long‐range electrostatic steering effects since the positive charge of M180K is beyond the cut‐off distance of 13 Å from acidic residues on the DH domain. Thus, we propose a second mechanism to explain the effects of M180K: stabilization of the IET‐competent closed state via hydrogen bonding of the ε‐amino group of M180K to the carboxylate group of the heme *b* propionate A moiety (Figure 3). In the closed state, the propionate A moiety of heme *b* and DH domain residues W295, S298, M309, and R689 form the propionate‐docking site (Tan et al., 2015). Given its close proximity of 3–4 Å to the carboxylate group of the heme *b* propionate, M180K is well positioned to contribute a hydrogen bond to this network (Table S2). An additional hydrogen bond would contribute to formation and stability of the CYT/DH closed state, which could explain the increased IET rates of the M180K variant.

Altogether, while the mechanisms of electrostatic steering could explain the improvements for most variants, the mechanism of heme *b* cofactor stabilization seems to explain the effects observed for M180K.

### 2.6 Multi‐site variants had synergistic effects on shifting the pH optimum and enhancing electron transfer

**FIGURE 4:**
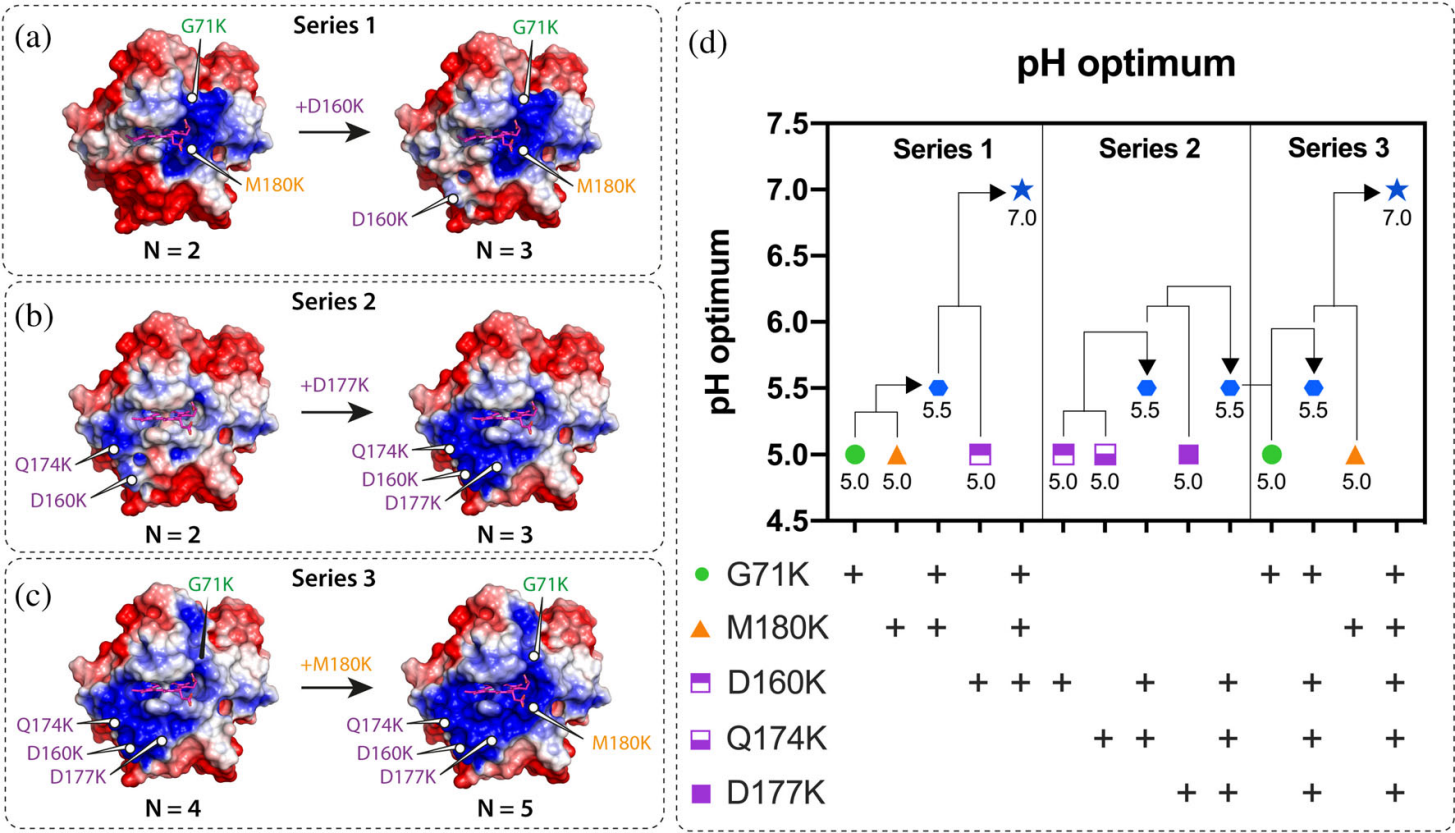
Combinatorial mutagenesis of interface positions shifted the pH optimum toward the neutral range. (a–c) Protein surface electrostatics of the CYT domain at pH 7.5 for variants of Series 1 (a), Series 2 (b), and Series 3 (c) calculated with APBS. The number of mutations (N) is indicated for each variant. The color code from Figure 2 is used. The electrostatic potential is shown as color gradient from red to blue (−2 to +2 k_B_T/e). Note that the charges of the heme *b* cofactor are not shown here. (d) The pH optimum of variants was measured with the cyt *c* assay in the presence of 30 mM lactose. Single‐site mutations G71K (green) from IR‐1, M180K (orange) from IR‐3, and D160K, Q174K, and D177K (purple) from IR‐2 were sequentially combined. Multiple variants (*blue*) are G71K/M180K, G71K/M180K/D160K, D160K/Q174K, D160K/Q174K/D177K, D160K/Q174K/D177K/G71K, D160K/Q174K/D177K/G71K/M180K. The pH optimum of the WT is at pH 4.5.

To further improve the pH optimum and IET rates of *Ch*CDH at pH 7.5, we sequentially combined the identified favorable mutations to create a series of combinatorial (or multi‐site) variants, which combine the three different interface regions and the two putative mechanisms of action (Tables 2 and S3, Figure 4). We hypothesized that combining different interface regions and mechanisms would improve IET rates more than focusing on only one interface region or mechanism. This effect should be apparent in both the pH optimum and the IET rates.

**TABLE 2:** pH optima and IET rates of combinatorial variants.^a^

		pH optimum		pH 4.5		pH 7.5	
Series	Variant	pH optimum	ΔpH versus WT	IET rate (s^−1^)	Fold change	IET rate (s^−1^)	Fold change
	WT	4.5	0	1.14 ± 0.08	1.0	0.10 ± 0.01	1.0
1	G71K, M180K	5.5	+1.0	1.09 ± 0.02	1.0	1.21 ± 0.03	12.1
	G71K, M180K, D160K	7.0	+2.5	0.5 ± 0.02	0.4	1.24 ± 0.05	12.4
2	D160K, Q174K	5.5	+1.0	0.07 ± 0.02	0.1	0.58 ± 0.17	5.8
	D160K, Q174K, D177K	5.5	+1.0	0.3 ± 0.01	0.3	0.48 ± 0.01	4.8
3	D160K, Q174K, D177K, G71K	5.5	+1.0	1.43 ± 0.13	1.3	0.06 ± 0.01	0.6
	D160K, Q174K, D177K, G71K, M180K	7.0	+2.5	0.7 ± 0.08	0.6	0.43 ± 0.05	4.3

^a^ Values are arithmetic means ± 95% confidence intervals of three independent experiments.

First, the most active variant M180K from IR‐3 (stabilizing the propionate docking site) was combined with G71K from IR‐1 and then D160K from IR‐2 (improving electrostatic steering effects) to investigate if the combination of all three CYT interface regions with two different mechanistic modes of actions would yield synergistic effects (Figure 4a, Series 1). Second, we combined the most promising mutations on IR‐2 into D160K/Q174K and D160K/Q174K/D177K to investigate the effects of accumulating positive charges at this hotspot for electrostatic steering (Figure 4b, Series 2). Third, the mutations from Series 1 and Series 2 were merged to create the quadruple variant D160K/Q174K/D177K/G71K and the quintuple variant D160K/Q174K/D177K/G71K/M180K (Figure 4c, Series 3), both of which should counterbalance all negative charges on the DH domain interface (Table S3).

Similar to the single‐site variants, the six multi‐site variants were produced and purified to homogeneity and their pH‐dependent IET rates were characterized (Figure S8). All combinatorial variants except D160K/Q174K/D177K/G71K showed a significantly improved pH optimum and IET rate at pH 7.5 over the WT (Figure 4d, Table 2). In Series 1, the combination of G71K and M180K shifted the pH optimum to pH 5.5 and increased the IET rate 12.1‐fold over the WT. Addition of D160K to G71K/M180K synergistically shifted the pH optimum to pH 7.0 and led to a 12.4‐fold increase in IET rate pH 7.5 compared to the WT.

The hypothesis that the synergistic effect is to be highest when combining variants from different interface regions is supported by two observations from Series 2 and 3. First, only a marginal effect on pH optimum and IET rate was observed in Series 2 where only IR‐2 mutations were combined. However, when adding M180K from IR‐3 to the quadruple mutant D160K/Q174K/D177K/G71K, the synergistic effect shifted the pH optimum to 7.0. We also observed synergistic effects for the increase in IET rates, but only to the point where three mutations were combined. The most active multi‐site variants at pH 7.5 were G71K/M180K (1.21 s^−1^) and G71K/M180K/D160K (1.24 s^−1^), equivalent to a 12.1‐ and 12.4‐fold enhancement over the WT (Figure 4d, Table 2). The D160K/Q174K/D177K/G71K variant had a lower IET rate (0.06 s^−1^) than the WT at pH 7.5 but the insertion of M180K increased the IET rate again (0.43 s^−1^) over the WT levels.

To conclude this section, three key findings can be defined: First, combining different interface regions is crucial to obtain optimal effects for the shift of the pH optimum and the increase in the IET rate at pH 7.5. Second, an accumulation of more than 3 mutations resulted in no further improvements in the IET rate at pH 7.5, even though the pH optimum was shifted even further toward neutral pH. One possible explanation for this finding is that the binding of the CYT to the DH domain may be too strong, inhibiting adoption of the open state to transfer electron to the terminal electron acceptor. Third, the combinatorial studies highlight the relevance of M180K as a crucial variant that stabilizes the interaction of the heme *b* propionate with its binding site at the DH domain.

### 2.7 Electrochemical characterization of the interface engineered variants

**FIGURE 5:**
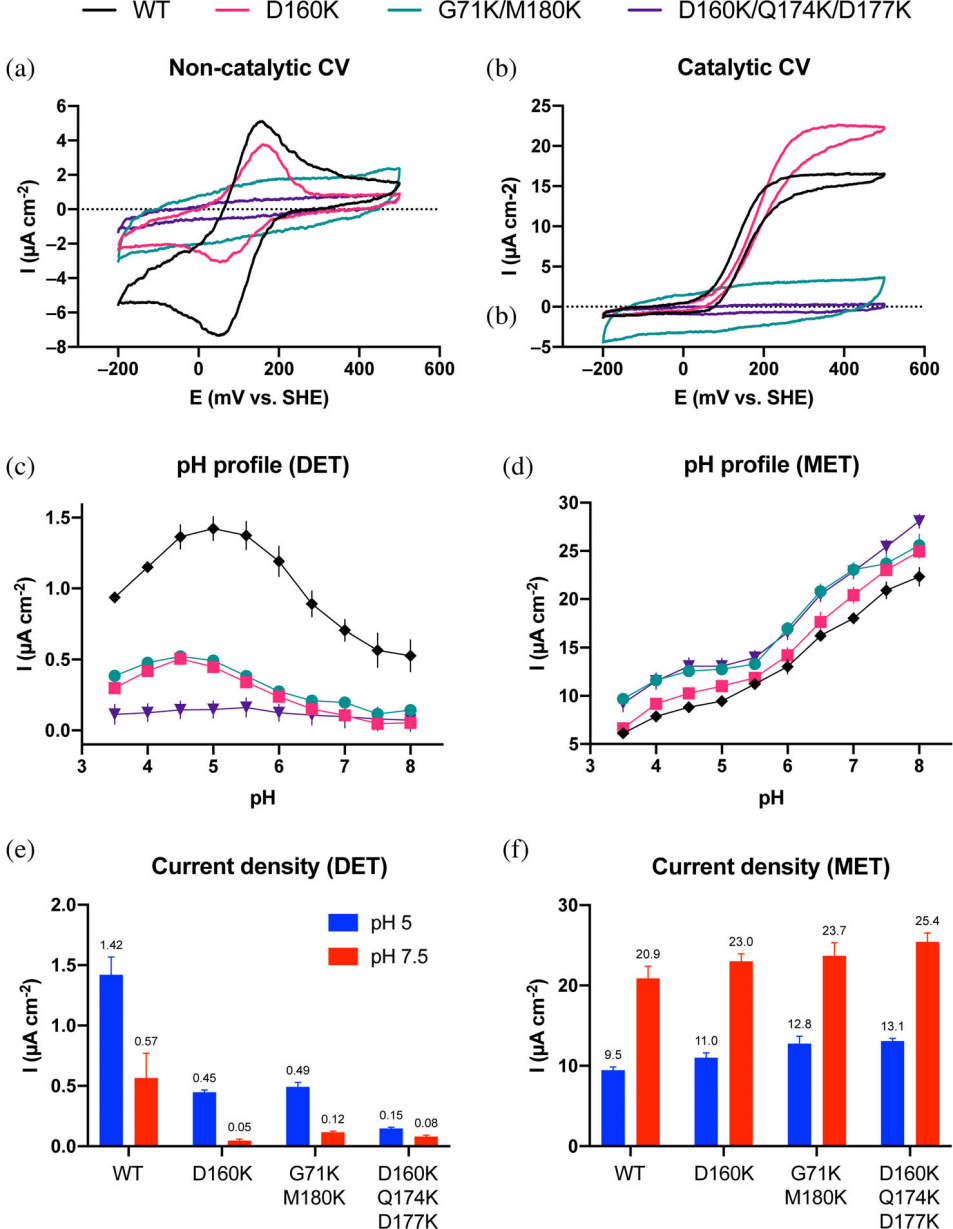
Electrochemical characterization of *Ch*CDH‐WT and three variants. (a, b) Cyclic voltammograms at non‐catalytic (a) and catalytic conditions (5 mM lactose, b) of WT *Ch*CDH (*black*), D160K (*magenta*), G71K/M180K (*green*), and D160K/Q174K/D177K (*purple*). Measurements were performed on aldrithiol‐modified gold electrodes at a scan rate of 10 mV s^−1^ in 50 mM citrate buffer pH 5.0 supplemented with 0.1 M KCl. The second of three recorded cycles is shown. (c, d) pH Profiles determined by flow‐injection analysis of CDH modified graphite electrodes in the DET (C) and MET mode (d) with 20 μM 1,4‐benzoquinone as redox mediator. (e, f) Current densities at pH 5.0 (*blue*) and pH 7.5 (*red*) for DET (e) and MET (f). Values are arithmetic means ± the standard error calculated from triplicate measurements of independently prepared electrodes.

To judge the usefulness of our improved variants for biosensor and biofuel cell applications, we performed electrochemical characterization in the DET and MET mode using cyclic voltammetry (Figure 5a,b, Figure S9) and amperometric flow injection analysis (Figure 5c–f). We hypothesized that the improvements observed in IET for our variants would translate into higher current densities in the DET mode while the MET currents would remain unchanged, given that the DH domain was not modified in this study and the enzyme is still functionally active.

First, cyclic voltammetry on thiol‐modified gold electrodes was performed. In the absence of substrate, cyclic voltammetry revealed a reduced electrode communication for all three variants compared to the WT as the peak couples attributable to the direct reduction and oxidation of the CYT domain is less pronounced for D160K or absent for G71K/M180K and D160K/Q174K/D177K (Figure 5a). The peak potential for D160K remained unchanged compared to the WT, indicating no significant structural changes around the heme *b* cofactor, which could modulate the redox potential. In the presence of 5 mM lactose as substrate, a distinct catalytic current with an onset potential attributable to the redox potential of CYT for the WT and variant D160K were observed, providing evidence for DET (Figure 5b). The combinatorial variants G71K/M180K and D160K/Q174K/D177K did not show a catalytic current, indicating that an accumulation of positive charges on the CYT domain disturbs the DET to the aldrithiol‐modified gold electrode.

Second, the pH profiles were assessed using amperometric flow injection analysis employing adsorbed WT and variants on graphite electrodes, which were polarized at +250 mV versus Ag|AgCl. DET and MET (1,4‐benzoquinoneone) currents were monitored upon successive injections of 5 mM lactose over a pH range of 3.5–8.0. DET currents originate from the electron transport chain from the catalytic DH domain to the heme domain (via the IET) to the electrode surface. MET responses additionally contain the extra current obtained from the shuttling of electrons from the DH domain directly to the electrode surface using the mediator. The pH profiles measured in the DET mode (Figure 5c) revealed a decreased current for all variants, retaining at pH 5.0 and 7.5, respectively, 25% and 8% (D160K), 28% and 21% (G71K/M180K), and 12% and 14% (D160K/Q174K/D177K) of the WT response (Figure 5e) and no shift of the pH profiles toward the neutral range. The decrease in current density was modified in a dose‐dependent manner depending on the number of positive charges introduced on the CYT domain (Figure S10). Apparently, the positive charged mutations at the CYT interface reduced the DET current on graphite electrodes for all variants, even though variant D160K worked well on gold electrodes. In contrast, pH profiles measured in the MET mode (Figure 5d) showed comparable currents to the WT for all variants, indicating that the catalytic activity is not compromised by the mutations (Figure 5f). MET currents are more than 10‐fold higher than DET currents, suggesting that only a fraction of the enzymes is in close contact with the electrode surface and oriented in a favorable manner enabling DET. As opposed to the DET currents, the MET currents are highest at pH 8.0 for all tested variants, which shows the potential to improve IET and DET for neutral or slightly alkaline pH.

Altogether, the electrochemical results demonstrate that accumulating positive charges on the CYT domain interface hampers DET to the aldrithiol‐modified gold electrode and the spectroscopic graphite electrode surface but not the catalytic activity or IET of the enzyme itself.

## 3 DISCUSSION

The results demonstrate that interface engineering of surface charges of *Ch*CDH is a suitable strategy to neutrally shift the pH optimum and substantially improve the IET rate (Figure 1). In light of previous research, this work strengthens the hypothesis that electrostatic repulsion of negative interface charges disrupt the formation of the closed state and thus decelerate IET of *Ch*CDH at neutral pH (Kadek et al., 2017, 2015; Kracher et al., 2015). Our results are also in line with the bridging effect of divalent metal cations, which is hypothesized to improve IET at neutral pH by neutralizing the negative charge repulsion between the CYT and DH domain (Schulz et al., 2012). As such, our interface‐engineered variants exhibit similar IET‐enhancing effects as the bridging effect of divalent metal cations, which can enhance the activity of *Ch*CDH up to 5‐fold (Schulz et al., 2012). However, in contrast to divalent cations, engineered *Ch*CDH variants can be used in continuous measurement systems such as CGM sensors (Jayakumar et al., 2022).

The fact that 15 of 17 single‐site mutants and all six multi‐site mutants were active and five variants showed shifted pH optima and several‐fold improved IET rates over the WT provides strong evidence for the feasibility of our interface engineering strategy (Figure 2, Table 1). Even though engineering of surface charges has been demonstrated to be a valuable tool to engineer improved enzymes (Pedersen et al., 2019), the major challenge of interface engineering in our case was to define and analyze the interface to select suitable targets, which is difficult because one is operating in a relatively small and confined space. As such, the probability of impairing the interface is much higher than the likelihood of improving it, as is often found for rational enzyme design (Kirk et al., 2018). To overcome this challenge, we rationally defined and analyzed the interface by applying a python script (interfaceResidues.py) on the high‐resolution structure of *Ch*CDH in the closed state (PDB: 4QI6) to acquire a high‐quality and objective set of interface residues. We then combined structure and sequence analyses to select interface target residues that, given their position adjacent to acidic residues on the DH domain interface and the presence of alternative alkaline amino acids in the MSA, would likely shift the pH optimum toward pH 7.5 and improve IET when replaced with alkaline residues such as lysine or arginine. Overall, replacing acidic with alkaline amino acids is often done in studies using rational design to engineer the pH‐dependent activity of enzymes, achieving pH optimum shifts of 0.5–1.5 logs and activity improvements of 2 to 9‐fold (Ito et al., 2021; Li et al., 2014; Ma et al., 2016; Pokhrel et al., 2012; Qiu & Lai, 2013; Turunen et al., 2002). For instance, one recent study used surface electrostatic models to engineer the surface charges of a glucose dehydrogenase–heme *b* domain fusion enzyme (capable of IET and DET), yielding two single‐site mutants with 1.7‐ und 9.0‐fold increased IET (Ito et al., 2021). In our study, we achieved similar effects for our single‐site variants but even greater enhancements for our combinatorial variants, shifting the pH optimum by up to 2.5 pH units and increasing IET by up to 12.4‐fold.

For most of the variants (except M180K), we hypothesize that improved surface charge compatibility and electrostatic steering can explain the observed improvements. Electrostatic steering is based on the theory of long‐range electrostatic interactions, which are weakly specific forces conferred by surface charges (Sheinerman et al., 2000) and play a major role in enzyme catalysis (Warshel et al., 2006) and protein–protein interactions (Vascon et al., 2020) such as antibody Fc heterodimer formation (Gunasekaran et al., 2010) and chaperone binding to their client proteins (Koldewey et al., 2016). As such, electrostatic steering can control the diffusion‐controlled speed and overall rate constant of protein–protein interactions (Alsallaq & Zhou, 2008). Therefore, interfaces with complementary charge distributions can lead to kinetic association constants that are substantially larger than uncomplimentary interfaces (Schreiber et al., 2009). The fact that electrostatic steering can be an effective way to shift the pH optimum and enhance enzymatic activity has also been reported in a previous study using rational design to engineer the pH optimum of xylanase (Pokhrel et al., 2012). Further, the electrostatic steering hypothesis agrees with a previous study that investigated the intermediates states of CDH complex formation using neutron crystallography and small‐angle x‐ray scattering, finding that *Ch*CDH populates various intermediate states between the closed and open conformation dependent on the surface electrostatics at different pH. (Bodenheimer et al., 2017). As such, electrostatic steering may decrease the likelihood of inactive intermediates and raise the probability of adopting the IET‐competent closed state in a more efficient manner.

Interestingly, we identified IR‐2 as a hotspot for pH profile engineering, given that all seven IR‐2 mutants showed a neutral shift in the pH optimum, and four IR‐2 variants showed higher IET rates than the WT (Figure 2b, Table 1). In general, interface hotspots are defined as clusters of residues that contribute most to a certain enzyme property and are thus often targeted for mutagenesis to improve this enzyme property (Qiao et al., 2018). In this regard, IR‐2 of *Ch*CDH can be considered a hotspot region. In a previous study using a similar sequence homolog‐based engineering strategy, mutation sites that improved the pH optimum of xylanase also clustered at a certain region of the enzyme (Ma et al., 2016). The identification of hotspots as targets for mutagenesis is beneficial as it leads relatively small libraries with a high percentage of positive hits (van der Meer et al., 2016). However, in our combinatorial mutagenesis studies, we found that clustering mutants at the supposed IR‐2 hotspot did not substantially improve the pH optimum or IET rate of *Ch*CDH compared to single‐site IR‐2 variants (Figure 4, Tables 1 and 2). Instead, only the combination of mutants from all three interface regions synergistically improved the pH optimum and IET rate. These findings suggest that many mutations spread across the entire CYT interface are needed to yield pH optimum shifts of >2 pH logs and IET improvements of >10‐fold.

In this regard, we hypothesize that M180K, given its proposed mechanism of stabilizing the heme *b* cofactor in the propionate docking site of the closed state (Figure 3), contributes mostly to the synergistic improvements seen in the combinatorial variants (Figure 4). The propionate‐docking site is essential for adoption of the IET‐competent closed state and it has been shown that mutations in this region can accelerate or decelerate the IET rate of *Ch*CDH (Tan et al., 2015). In contrast, the wildtype methionine at this position cannot form a hydrogen bond with the propionate A carboxylate group, but only participate in Van‐der‐Waals and hydrophobic interactions or sulfur‐aromatic interactions with the heme porphyrin ring (Aledo, 2019). The pH‐shifting and IET‐improving potential of M180K is most evident in the combinatorial mutagenesis studies (Table 2, Figure 4). In each mutagenesis series, addition of M180K substantially improved the pH optimum and IET rate whereas insertion of other hit mutants showed only marginal effects. Altogether, these results suggest that stabilization of the heme *b* cofactor in the closed state is beneficial to improve the IET rate at neutral pH.

One limitation with our interface engineering approach was the disruption of DET to the hydrophobic gold and graphite electrode surfaces as more positive charges were accumulated on the CYT domain (Figures 5 and S10). Of note, the mutations did not alter the catalytic activity of *Ch*CDH, as is apparent in the biochemical studies and current densities in MET mode, which are independent of the CYT domain and unchanged compared to the WT (Figure 2f). We hypothesize that the introduced lysine mutations increase the charge and polarity of the CYT domain, which is beneficial for IET as it increases the affinity for the negatively charged DH domain but at the same time detrimental for DET as it decreases the affinity of the engineered CYT domain to the hydrophobic electrode surface. To overcome this problem, investigation of different electrode materials and surface modifications is required, as the electrochemical setup used in this study was initially developed for optimal performance of the *Ch*CDH‐WT operating at acidic pH. Alternatively, future work could focus on interface engineering of the DH domain to improve the pH optimum and IET rate while maintaining DET to electrode surfaces via the CYT domain.

## 4 CONCLUSION

CDH, with its built‐in redox mediator CYT, is an interesting enzyme for third‐generation CGM systems because its activity and IET can be modulated and adapted to different conditions by enzyme engineering. This study has shown that the acidic IET pH optimum of *Ch*CDH can be shifted toward neutral pH by engineering the domain interface while also increasing its IET rate. The variants achieve these enhancements through electrostatic steering, in which the charges of the engineered interface direct the CYT domain toward the DH domain, improving the kinetics of complex formation through long‐range electrostatic interactions. This IET‐competent closed state of *Ch*CDH can be further supported by a hydrogen bonding network involving M180K through stabilization of the carboxylate group of heme *b* propionate. Although the engineering of the domain interface to optimize the pH optimum and IET was successful, it could not be used to increase the DET from the CYT domain to the gold or graphite electrodes used in this study. The positive charges at the CYT interface either reduce or completely prevent the DET with the selected electrodes. To overcome this problem, other electrode materials and surface modifications should be investigated. Overall, this study is an important step toward understanding the domain interactions of CDH, but only a first step toward developing an optimized bioelectrocatalyst for third‐generation CGM systems.

## 5 MATERIALS AND METHODS

### 5.1 Chemicals

Unless stated otherwise, chemicals were obtained from Sigma Aldrich or Merck. Solvents and fermentation media components were purchased from Carl Roth. For electrochemical experiments, disodium citrate and sulfuric acid were purchased from BDH (Poole, GB) and sodium hydroxide from Labassco (Stockholm, SE). All chemicals were of analytical grade or the highest grade of purity available. Aqueous solutions were prepared with deionized water (>18 MΩ cm).

### 5.2 Strains, vectors, media, buffers, and solutions

*Komagataella phaffi* (formerly *Pichia pastoris*) strain X33 and the vector pPICZɑ were purchased from Invitrogen (Carlsbad, California, USA). *K. phaffii* strain X33 was maintained on YPD (10 g L^−1^ yeast extract, 20 g L^−1^ peptone, and 10 L^−1^ glucose) agar plates. All the transformants derived from X33 were maintained on YDP plates containing 100 μg mL^−1^ zeocin. Vector pPICZɑ and all cloned plasmids were prepared using the QIAprep Spin Miniprep kit (Qiagen, Valencia, California, USA). Restriction endonucleases, Phusion‐High‐Fidelity DNA Polymerase, and T4 DNA ligase were purchased from New England Biolabs (Ipswich, Massachusetts, USA). Oligonucleotide primers were synthesized by Microsynth (Balgach, Switzerland). Buffered‐Methanol‐Complex Medium (BMMY) used for protein expression in *K. phaffii* was prepared with the EasySelect *Pichia* Expression Kit (Invitrogen, Carlsbad, CA, USA).

### 5.3 Site‐directed mutagenesis

Cloning, sequence analysis, and heterologous expression of the thermostable CDH gene from *Crassicarpon hotsonii* (formerly *Myriococcum thermophilum*, CDH IIA; gene *cdh*; UniProt A9XK88) followed a published procedure (Zámocký et al., 2008). All variants were generated via a two‐step site‐directed mutagenesis. The single‐site mutations comprise G71K, K72 (lysine insertion after G71), G72K, N76K, T97K, W155K, W155R, Q157K, F159R, F159K, D160G, D160K, D160R, Q174K, D177K, M180K, and I182K. Combinatorial variants comprise G17K/M180K, D160K/Q174K, G71K/M180K/D160K, D160K/Q174K/D177K, D160K/Q174K/D177K/G71K, and D160K/Q174K/D177K/G71K/M180K. Clean‐up of PCR amplicons was performed via agarose gel electrophoresis using the Wizard SV Gel and PCR Clean‐Up System (Promega, Madison, WI, USA) according to the manufacturer's recommendations. Purified PCR amplicons of the mutated genes were cloned into the expression vector pPICZɑ (Invitrogen, Carlsbad, CA, USA) downstream of the alcohol oxidase I (AOXI) promotor. Ligated plasmids were transformed into *E. coli* strain BL21 using heat‐shock and grown on LB agar plates overnight. Plasmids were prepared by cultivating three independent clones overnight in LB medium and plasmid purification using the QIAprep Spin Miniprep kit (Qiagen, Valencia, California, USA). Plasmids were eluted using deionized Milli‐Q water for storage. Plasmid integrity and all mutations were confirmed by sequencing using the conventional forward and reverse *AOXI* sequencing primers. Plasmids with a verified sequence were stored at −80°C until further usage.

### 5.4 Recombinant production of CDH variants

*K. phaffii* X33 competent cells were prepared by following the procedure described in the Easy Select Pichia Expression System manual from Invitrogen. The recombinant pPICZ plasmids were linearized for 1 h using the restriction endonuclease *PmeI* and transformed into *K. phaffii* X33 competent cells via electroporation using the MicroPulser Electroporator from Bio‐Rad Laboratories (Hercules, CA, USA) according to the manufacturer's manual. Transformations were then selected on YPD agar (20 g L^−1^ yeast extract, 40 g L^−1^ peptone, 20 g L^−1^ dextrose, and 40 g L^−1^ agar) plates containing 100 μg mL^−1^ zeocin (Biobasic, USA). Successful genome integration of recombinant pPICZ plasmids was confirmed by colony PCR using forward and reverse AOX primers. Verified clones of variants were first cultivated in 150 mL baffled shaking flasks containing 50 mL YPD with 100 μg mL^−1^ zeocin overnight at 30°C in a shaking incubator at 110 rpm until the optical density at 600 nm (OD_600_) reached 10–15. Main cultures in 1‐L baffled flasks were inoculated to an OD_600_ of 1 in 200 mL of YPD medium containing 1.2% (v/v) methanol to induce AOX‐inducible recombinant protein production. The incubation was performed at 30°C and 110 rpm in a shaking incubator. Monitoring of main cultures was performed via OD_600_ measurements and determination of the enzymatic cytochrome *c* (cyt *c*) activity assays using centrifuged culture supernatants. The total duration of protein production depended on the observed enzymatic activity and OD_600_ of the main cultures. The supernatant containing the secreted recombinant enzyme variant was cleared by centrifugation at 10,000 rpm for 30 min at 4°C. The clear supernatant was then concentrated and diafiltrated with water using a polyethersulfone hollow‐fiber crossflow module with a 10‐kDa cut‐off (Microza UF module SLP‐1053; Pall Corporation) until a conductivity of 2–3 mS cm^−1^ was obtained. The concentrated enzyme solution was stored at 4°C until chromatographic purification.

### 5.5 Chromatographic purification of variants

Enzyme purification was performed using hydrophobic interaction chromatography (HIC) and anion exchange chromatography (AIEX) as previously established for *Ch*CDH (Zámocký et al., 2008). The purification was performed on an ÄKTA Pure FPLC system (GE Healthcare). The concentrated enzyme solution was first loaded onto a Phenyl‐Source column (HR 26/20 from GE Healthcare) pre‐equilibrated with 50 mM sodium acetate buffer, pH 5.5, containing 0.2 M NaCl and ammonium sulfate (20% saturated). Protein elution was achieved by a linear gradient against the elution buffer (50 mM sodium acetate buffer, pH 5.5). Fractions with the highest specific CDH activity were pooled, concentrated, and diafiltrated as described above to a conductivity of 5 mS cm^−1^ in 50 mM sodium acetate buffer, pH 5.5. The concentrated enzyme solution was loaded onto a Q‐Source column (HR 26/20 GE Healthcare) pre‐equilibrated in 50 mM sodium acetate buffer, pH 5.5. Proteins were eluted by a linear gradient of 50 mM sodium acetate buffer, pH 5.5, containing 0.5 M NaCl. Fractions with the highest CDH activity were pooled, concentrated, and diafiltered using Amicon Ultra‐15 Centrifugal Filters (30 kDa cut‐off). The protein concentration was determined using the absorbance at 280 nm and the specific molar absorption coefficient of *Ch*CDH (ε_280_ = 159 mM^−1^ cm^−1^) calculated with ProtParam (Gasteiger et al., 2003). Enzyme solutions were stored at 4°C.

### 5.6 Enzyme activity assay

The activity of wild type (WT) *Ch*CDH and variants were spectrophotometrically determined by the reduction of the electron acceptor cytochrome *c* (cyt *c*, ε_550_ = 19.6 mM^−1^ cm^−1^) in presence of 30 mM lactose as substrate (Baminger et al., 2001). The cyt *c* assay measures the IET rate since cyt *c* is specifically reduced by the CYT domain, which can only occur after substrate oxidation in the DH domain and the rate‐limiting IET to the CYT domain. Reactions were monitored for 180 s at 30°C in a Lambda 35 UV–Vis spectrophotometer (Perkin Elmer) performed with 20 μM cyt *c* at 520 nm in McIlvaine buffer (McIlvaine, 1921). Measurements were done in triplicates. The relative standard deviations of replicates were <10%. Volumetric activities were calculated according to the Lambert–Beer law. IET rates were calculated from the protein concentration, the volumetric activity, and the theoretical molecular weight of the enzyme calculated via the ExPASy ProtParam tool (http://web.expasy.org/protparam) (Gasteiger et al., 2005).

### 5.7 Determination of pH profiles in solution

To determine pH activity profiles, the initial rates were measured at 30°C in McIlvaine buffer at pH 3.0–8.5 with 30 mM lactose (saturating conditions) using the cyt *c* assay. Purified enzymes were used at a concentration of 50 μg mL^−1^. Measurements were done in triplicates.

### 5.8 Statistical analysis

Statistical analysis was performed in Excel for Mac Version 16.48 (Microsoft®) and Prism 8 Version 8.4.0 for macOS (GraphPad Software, San Diego, California USA). All investigated parameters are expressed as arithmetic means ± the 95% confidence intervals of three independent experiments unless stated otherwise. Enzymatic activity measurements were performed in triplicates. Normality of distribution was tested using the Kolmogorov–Smirnov test (Berger & Zhou, 2014). We assessed differences between the variants and the WT using parametric one‐way analysis of variance (1‐way ANOVA) (Heiberger & Neuwirth, 2009) followed by Fisher's Least Significant Difference (LSD) test. Differences with *p* < 0.05 were considered statistically significant. Asterisks were used in the figures to indicate the statistical significance with *p* < 0.0001 (***), *p* < 0.001 (**), and *p* < 0.05 (*). We did not correct for multiple comparisons via post hoc testing to minimize false‐negative rates due to the limited number of variants (Rothman, 1990; Saville, 1990). To assess the different effects of variants on the IET rates and pH optima, the covariations between relevant parameters were assessed using Pearson correlation analysis and expressed as the Pearson correlation coefficient (*r*).

### 5.9 Protein surface electrostatics

For the generation of surface charged‐based pH profiles, we used the pHmap software (https://github.com/riccstick/pHmap) (Breslmayr, 2021). The CYT and DH domain structures were individually extracted from the crystallographic structure of full‐length *Ch*CDH (PDB: 4QI6) (Tan et al., 2015). Hydrogen atoms were assigned to the structures at pH 5 and pH 7.5 using the PROPKA algorithm (Olsson et al., 2011) combined with the PDB2PQR (Dolinsky et al., 2004) Version 2.1.1. The FAD and heme *b* cofactors were also included as extracted MOL2 files from the crystal structure. Visualization of surface electrostatics was performed using the Adaptive Poisson‐Boltzmann (APBS) (Jurrus et al., 2018) software package Version 3.0.0 combined with the PyMOL Molecular Graphics System Version 2.3.4 (Schrödinger, LLC.) with the APBS Tools 2.1 plugin. The electrostatic potential is shown as color gradient from red to blue (−2 to +2 k_B_T/e) unless stated otherwise.

### 5.10 Structural analysis of interface residues

The interface of CDH is defined as the residues that become buried after transition from the open (PDB: 4QI7) to the closed conformation (PDB: 4QI6) (Tan et al., 2015). Interface residues were identified using the python script interfaceResidues.py, which can be downloaded by accessing PyMOL wiki (https://pymolwiki.org/index.php/InterfaceResidues). These interfacial residues were then analyzed with the PyMOL Molecular Graphics System Version 2.3.4 (Schrödinger, LLC.).

### 5.11 Sequence analysis and phylogeny

We performed multiple sequence analysis (MSA) and phylogenetic analysis to investigate the sequence homology and conservation rate of our selected positions from the structural analysis. Sequence mining was performed using the Hmmer algorithm phmmer (Potter et al., 2018) with *Neurospora crassa* CDH IIB (GI 3874381) as a template. Hits were filtered by taxonomy and domain architecture, resulting in 362 putative CDH sequences (317 ascomycetous and 45 basidiomycetous sequences). Sequences without a cytochrome motif were omitted. The ClustalO algorithm (Sievers & Higgins, 2014) was used to construct an MSA. Phylogenetic analysis of the MSA was performed using Randomized Axelerated Maximum Likelihood‐Next Generation (Kozlov et al., 2019). The best‐fit substitution model for amino acid sequence “ModelTest‐NG” was used (Darriba et al., 2020). The Wheelan and Goldman model (Whelan & Goldman, 2001) with frequencies, invariant sited, the number of gamma‐distributed sites set to 4 was chosen, and 20 starting trees were calculated for tree inference. A standard nonparametric bootstrap analysis was carried out until convergence criteria (cut‐off 0.03) were reached based on the bootstrapping test (Pattengale et al., 2010). The bootstrapped trees were mapped onto the best‐scoring most likelihood tree for branch support visualization on the original multiple sequence alignment.

### 5.12 Cyclic voltammetry

Preparation of gold electrodes (BASi, West Lafayette, IN, USA; Ø = 1.6 mm) started with the oxidative cleaning by immersion in acidic Piranha solution (3:1 mixture (v/v) of concentrated H_2_SO_4_ and 30% H_2_O_2_) for 3 min and subsequent rinsing with copious amounts of Milli‐Q water (Caution! Piranha solution is extremely energetic and may result in explosion. Handle under the most cautious circumstances). Electrodes were mechanically polished in 0.1 μm alumina polishing suspension (Struers GmbH, Willich, DE) on a polishing microcloth (Buehler, Lake Bluff, IL, USA), rinsed with Milli‐Q water, and ultrasonicated for 5 min. Electrodes were electrochemically cleaned by cycling in 0.5 M H_2_SO_4_ (20 cycles, 300 mV s^−1^, −0.1 to +1.7 V vs. Ag|AgCl [saturated KCl]), followed by a final rinsing step with Milli‐Q water. Clean gold electrodes were immersed in a 1 mM aldrithiol solution (in EtOH, 96%) for 1 h to form a self‐assembled monolayer. Electrodes were rinsed with 20% EtOH and Milli‐Q water to wash away unbound thiols. Cleaned electrodes were dried under a stream of nitrogen. A total of 2 μL enzyme solution were drop casted onto the self‐assembled monolayer modified gold electrode surface and dried at room temperature. The dried droplet was covered with a presoaked (in buffer) dialysis membrane (Rancho Dominguez, CA, USA, regenerated cellulose, 12 kDa cut‐off) held in place with a rubber O‐ring. Cyclic voltammetry was carried out using a standard three‐electrode setup with the enzyme‐modified gold electrodes as working electrode, an Ag|AgCl (saturated KCl) electrode as a reference, and a platinum wire as counter electrode in argon saturated (15 min purging) 50 mM sodium citrate buffer, pH 5.0, supplemented with 0.1 M KCl, at scan rates of 10, 20, 50, and 100 mV s^−1^ between −0.35 and +0.3 V versus Ag|AgCl. To examine catalytic currents, 5 mM lactose was added to the bulk.

### 5.13 Flow injection analysis

Preparation of spectroscopic graphite electrodes started with mechanical polishing of graphite rods (SGL Carbon GmbH Werk Ringsdorff, Bonn, DE; Ø = 3.05 mm) on wetted emery paper and subsequent rinsing with Milli‐Q water. *Ch*CDH variants were set to a final volumetric activity of 18 U mL^−1^. A volume of 5 μL enzyme solution (in 50 mM sodium citrate buffer, pH 5.0) was drop casted onto the electrode surface and dried at room temperature. Immobilization via chemo‐physical adsorption was completed by incubating the modified electrodes at 4°C overnight. Amperometric flow injection analysis was carried out using a standard three‐electrode setup with the enzyme‐modified graphite electrode as the working electrode, an Ag|AgCl (0.1 M KCl) reference, and a platinum wire counter electrode connected to a potentiostat (Zäta Elektronik, Höör, SE). The enzyme‐modified graphite electrode was press‐fitted into a homemade wall jet cell connected to a flow injection system comprising a LabPro six‐port injector (Rheo‐dyne, Cotatit, CA, USA) with a 50 μL injection loop and a Minipuls 2 peristaltic pump (Gilson, Middleton; WI, USA). The injection loop was used to inject 5 mM lactose (DET measurements) or 5 mM lactose and 20 μM 1,4‐benzoquinone (BQ, MET measurements). The peristaltic pump was used to apply a constant laminar flow of running buffer in the DET mode or running buffer with BQ for the MET mode across the enzyme‐modified working electrode. Applied running buffers were 50 mM sodium citrate (pH 3.5–7.0) or 50 mM disodium hydrogen phosphate (pH 6.5–8.0). Amperometric currents were recorded at 0.25 mV versus Ag|AgCl (0.1 M KCl). All variants were measured in triplicates of independent enzyme‐modified electrodes.

## AUTHOR CONTRIBUTIONS

**Thomas M. B. Reichhart:** Data curation (lead); formal analysis (lead); methodology (lead); visualization (lead); writing – original draft (equal); writing – review and editing (equal). **Stefan Scheiblbrandner:** Data curation (equal); formal analysis (equal); methodology (equal); visualization (equal); writing – original draft (equal); writing – review and editing (equal). **Christoph Sygmund:** Investigation (equal); methodology (equal). **Wolfgang Harreither:** Investigation (equal); methodology (equal). **Josef Schenkenfelder:** Investigation (equal); methodology (equal). **Christopher Schulz:** Investigation (equal); methodology (equal); writing – original draft (equal); writing – review and editing (equal). **Alfons K. G. Felice:** Investigation (equal); methodology (equal). **Lo Gorton:** Conceptualization (equal); funding acquisition (supporting); resources (supporting); supervision (supporting); writing – original draft (equal); writing – review and editing (equal). **Roland Ludwig:** Conceptualization (equal); funding acquisition (lead); project administration (lead); resources (lead); supervision (lead); writing – original draft (equal); writing – review and editing (equal).

## CONFLICT OF INTEREST STATEMENT

Thomas M.B. Reichhart reports a relationship with DirectSens GmbH that includes employment. Christoph Sygmund reports a relationship with DirectSens GmbH that includes board membership and equity or stocks. Wolfgang Harreither reports a relationship with DirectSens GmbH that includes board membership and equity or stocks. Christopher Schulz reports a relationship with DirectSens GmbH that includes employment. Alfons K.G. Felice reports a relationship with DirectSens GmbH that includes board membership and equity or stocks. Roland Ludwig reports a relationship with DirectSens GmbH that includes board membership and equity or stocks.

## Supporting information


**Table S1.** Interface residues of CDH within the CYT/DH complex.
**Table S2.** Charge‐to‐charge distances (in Å) of mutants and charged CYT/DH interface residues.
**Table S3.** Putative long‐range electrostatic forces of multi‐site variants.
**Figure S1.** Structure of the CYT/DH interface of *Ch*CDH.
**Figure S2.** Interface residues of the CYT domain of *Ch*CDH.
**Figure S3.** Analysis of amino acid conservation at selected positions.
**Figure S4.** Proof‐of‐concept on position D160.
**Figure S5.** Determination of cyt *c* assay threshold.
**Figure S6.** pH profiles of single‐site variants.
**Figure S7.** Correlation analysis of electron transfer rates and pH optimum.
**Figure S8.** pH Profiles of multi‐site variants.
**Figure S9.** Cyclic voltammograms of WT *Ch*CDH and D160K.
**Figure S10.** Correlation of charge change of variants with current density in DET mode.

## Data Availability

All data will be made available upon request.
