# Supplementary material for: Interface engineering of cellobiose dehydrogenase improves interdomain electron transfer
Source: Protein Sci. 2023 Aug 1;32(8):e4702. doi: 10.1002/pro.4702 (PMC10357501; doi:10.1002/pro.4702)
Supplement: Supplementary file 1 — Table S1. Interface residues of CDH within the CYT/DH complex. Table S2. Charge‐to‐charge distances (in Å) of mutants and charged CYT/DH interface residues. Table S3. Putative long‐range electrostatic forces of multi‐site variants. Figure S1. Structure of the CYT/DH interface of ChCDH. Figure S2. Interface residues of the CYT domain of ChCDH. Figure S3. Analysis of amino acid conservation at selected positions. Figure S4. Proof‐of‐concept on position D160. Figure S5. Determination of cyt c assay threshold. Figure S6. pH profiles of single‐site variants. Figure S7. Correlation analysis of electron transfer rates and pH optimum. Figure S8. pH Profiles of multi‐site variants. Figure S9. Cyclic voltammograms of WT ChCDH and D160K. Figure S10. Correlation of charge change of variants with current density in DET mode. [file PRO-32-e4702-s001.docx]

Supporting Information

Interface engineering of cellobiose dehydrogenase improves interdomain electron transfer

*Thomas M.B. Reichhart^1,2^, Stefan Scheiblbrandner^1^, Christoph Sygmund^,2^, Wolfgang Harreither^1,2^, Josef Schenkenfelder^1^, Christopher Schulz^2^, Alfons K.G. Felice^2^, Lo Gorton^3^, Roland Ludwig^1,2^**

^1^ Biocatalysis and Biosensing Laboratory, Department of Food Science and Technology, BOKU–University of Natural Resources and Life Sciences, Muthgasse 18, 1190 Vienna, Austria

^2^ DirectSens GmbH, Am Rosenbühel 38, 3400 Klosterneuburg, Austria

^3^ Department of Analytical Chemistry/Biochemistry, Lund University, P.O.Box 124, 22100 Lund, Sweden

* To whom correspondence should be addressed: Roland Ludwig, Department of Food Science and Technology, BOKU–University of Natural Resources and Life Sciences, Muthgasse 18, 1190 Vienna, Austria; E-mail: roland.ludwig@boku.ac.at; Telephone: +43 1 47654 75216

**ABBREVIATIONS**: CDH, cellobiose dehydrogenase; *Ch*CDH, CDH from *Crassicarpon hotsonii* (formerly *Myriococcum thermophilum)*; cyt *c*, cytochrome *c*; CYT, cytochrome domain; DET, direct electron transfer; DH, dehydrogenase domain; IET, interdomain electron transfer; IR, interface region; MET, mediated electron transfer; MSA, multiple sequence alignment; WT, wild type

# **Table S1. Interface residues of *Ch*CDH in the closed state.**

| **Type** | **Residue count** | **Percentage of total residues** | **Residues** ^a^ |
| --- | --- | --- | --- |
| Hydrophobic | 31 | 48% | CYT: **G71**, **G72**, P73, G98, A100, G138, G139, V152, **W155**, V156, A158, **F159**, G179, **M180**, G181, **I182**, W183, G184DH: W295, V296, A302, M309, L606, G607, A608, P609, P634, I639, V691, P694 |
| Polar | 25 | 39% | CYT: N3, Q28, T75, **N76**, **T97**, Y99, H137, S140, Q157, **Q174**, Q175, H176, N178DH: S298, C303, T306, Q308, S605, T633, S635, T637, S689, S693, S695, N696 |
| Ionizable | 8 | 13% | CYT: **D160**, **D177**DH: D297, K299, E304, D305, D307, R698 |

^a^ The interface of CDH is defined as the residues that become buried after transition from the open (PDB ID: 4QI7) to the closed conformation (PDB ID: 4QI6). Interface residues were identified using the python script interfaceResidues.py, which can be downloaded by accessing PyMOL wiki interfaceResidues (<https://pymolwiki.org/index.php/InterfaceResidues>). Residues selected for interface engineering based on structure and sequence analyses are shown in bold.

# **Table S2. Charge-to-charge distances (in Å) of mutants and ionizable interface residues.**^a^

| **Interface location** | | **Core interface residues** | | | | | | **Outer interface residues** | | | |  |  |
| --- | --- | --- | --- | --- | --- | --- | --- | --- | --- | --- | --- | --- | --- |
| **Region** | **Residue** | **D297** | **K299** | **E304** | **D307** | **R698** | **E275** | **D547** | **E550** | **D553** | **D642** | **heme^b^** | |
| **IR-1** | **G71K** | >13 | >13 | >13 | **9.1** | >13 | >13 | >13 | >13 | >13 | **10.3** | >13 | |
| **IR-2** | **D160K** | **9.1** | **9.7** | >13 | >13 | >13 | **12.2** | >13 | >13 | **10.9** | >13 | >13 | |
|  | **Q174K** | **9.2** | **5.2** | **12.4** | >13 | >13 | >13 | >13 | >13 | >13 | >13 | >13 | |
|  | **D177K** | **7.2** | **12.8** | >13 | >13 | >13 | **11.8** | **12.1** | **8.8** | **11.2** | >13 | **9.7** | |
| **IR-3** | **M180K** | >13 | >13 | >13 | >13 | **7.7** | >13 | >13 | >13 | >13 | >13 | **4.0** | |

^a^ Distances of >13 Å are shown in bold. All distances are shown in Ångstroms and were calculated in PyMOL using the mutagenesis and distance measurement wizard. Note that the distances may vary by ±1 Å depending on which of the lowest strain rotamers is chosen for the mutation. Here, we used one of the three lowest strain rotamers showing the shortest distance.

^b^Distance to the carboxylate group of the propionate A moiety of the heme *b* cofactor

# **Table S3. Putative long-range electrostatic forces of multi-site variants.^a^**

| **Interface location** | | **Core interface residues** | | | | | | **Outer interface residues** | | | |  |
| --- | --- | --- | --- | --- | --- | --- | --- | --- | --- | --- | --- | --- |
| **Series** | **Residues** | **D297** | **K299** | **E304** | **D307** | **R698** | **E275** | **D547** | **E550** | **D553** | **D642** | **heme^b^** |
| 1 | G71K, M180K |  |  |  | x | x |  |  |  |  | x | xx |
|  | G71K, M180K, D160K | x | x |  | x | x |  |  |  | x | x | xx |
| 2 | D160K, Q174K | x | x | x |  |  | x |  |  | x |  |  |
|  | D160K, Q174K, D177K | x | x | x |  |  | x | x | x | x |  | x |
| 3 | D160K, Q174K, D177K, G71K | x | x | x | x |  | x | x | x | x | x | x |
|  | D160K, Q174K, D177K, G71K, M180K | x | x | x | x | x | x | x | x | x | x | xx |

^a^ Residues marked with an “x” indicate a putative long-range electrostatic interaction. Residues with “xx” indicate a direct polar or electrostatic interaction for M180K with the propionate A carboxylate group of the heme *b* cofactor.


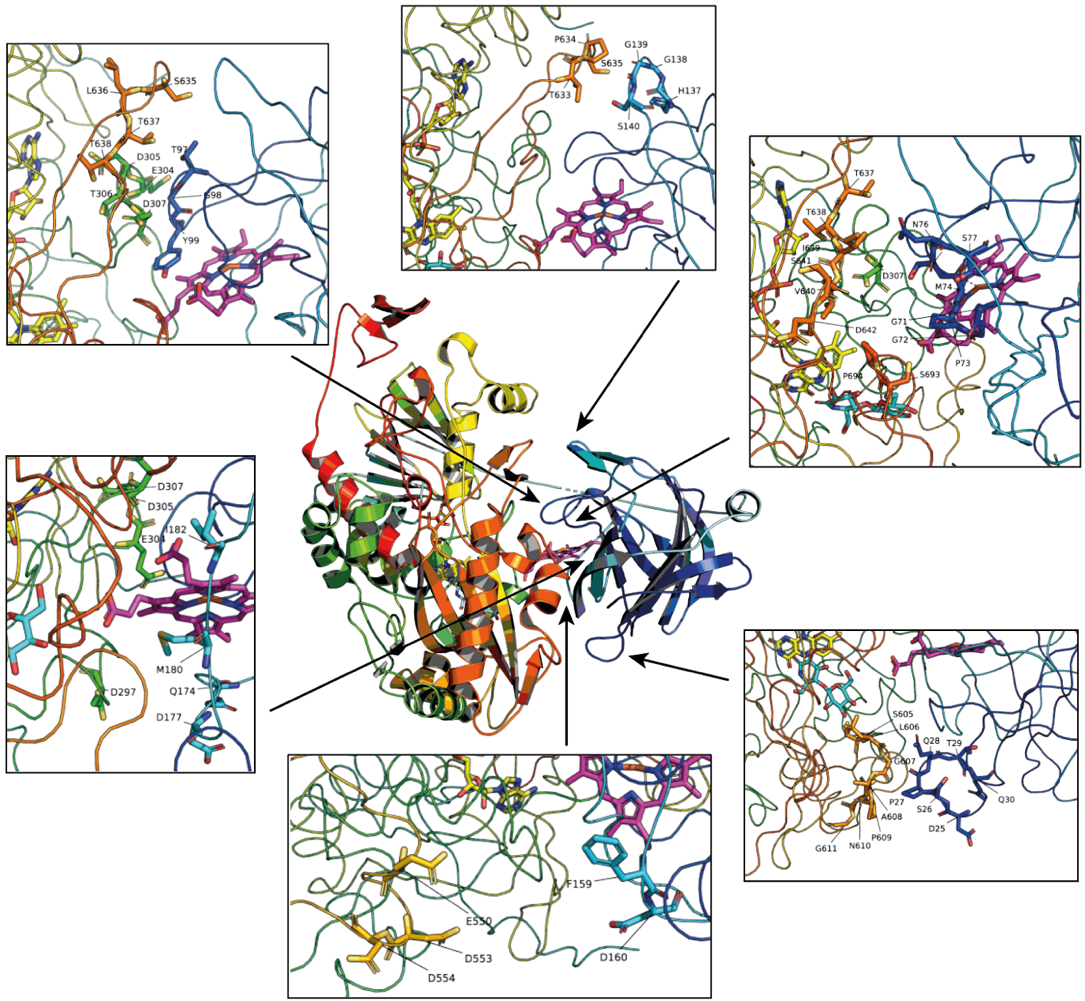


# **Figure S1. Structural analysis of the CYT/DH** **interface of *Ch*CDH.** The CYT/DH domain interaction interface was analyzed based on the full-length *Ch*CDH crystal structure (PDB ID: 4QI6). Relevant residues are labeled and shown as sticks. The FAD and heme *b* cofactor are also shown as yellow and magenta sticks, respectively. The co-crystallized cellobiono-1,5-lactame (from PDEB ID: 4QI5) was superimposed and is displayed as cyan sticks. This figure was prepared using PyMOL Molecular Graphics System (Version 2.3.4, Schrödinger, LLC.).

# **
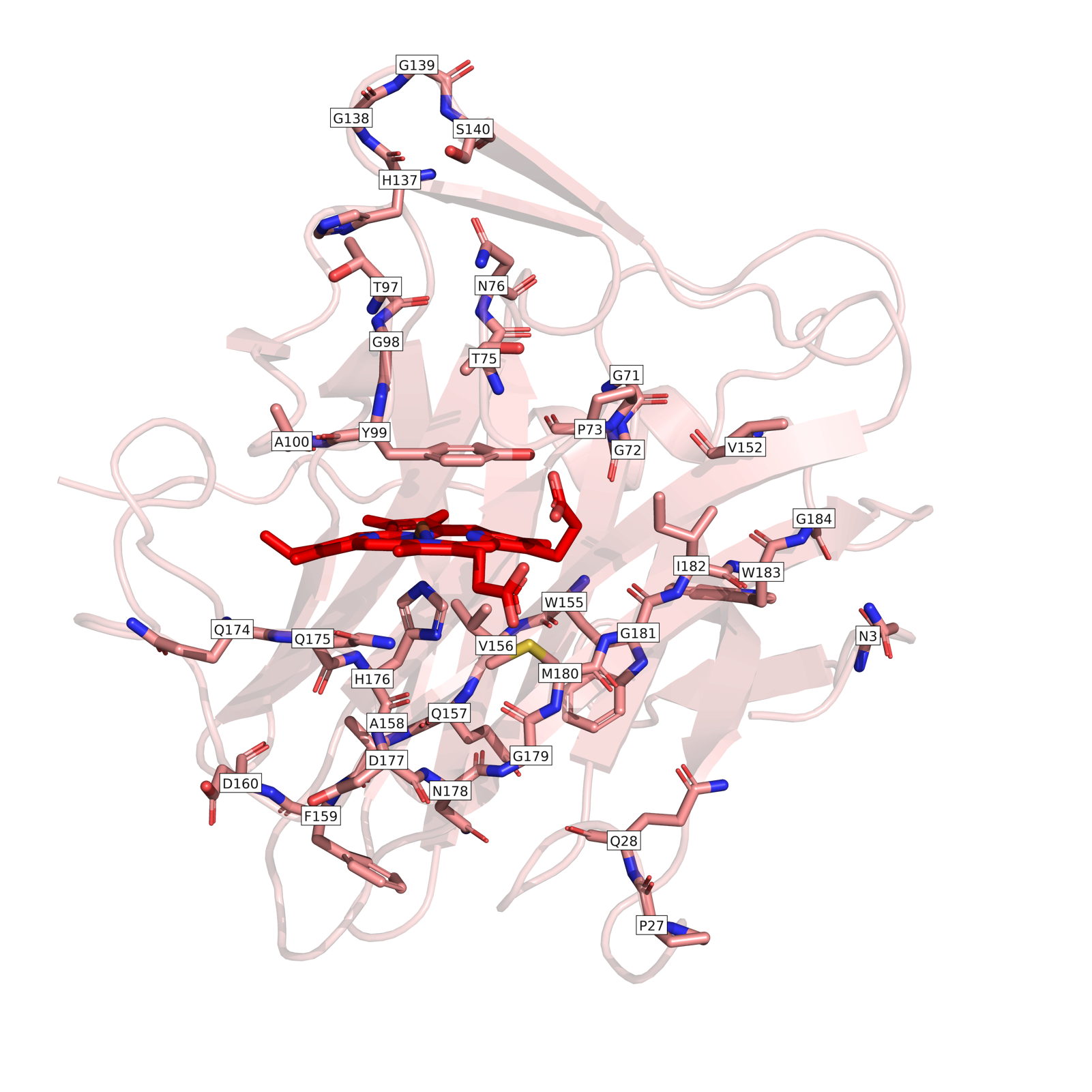
Figure S2 Interface residues of the CYT domain of *Ch*CDH.** The interface residues of the CYT domain (shown as cartoon) participating in the CYT/DH domain interaction were selected using the python script interfaceResidues.py (<https://pymolwiki.org/index.php/InterfaceResidues>) and are shown as sticks. The heme *b* cofactor is shown as red sticks. Note that the view of the CYT domain as displayed here resembles the interface region that faces the DH domain interface in the closed state (PDB ID: 4QI6). This figure was prepared using PyMOL Molecular Graphics System (Version 2.3.4, Schrödinger, LLC.).


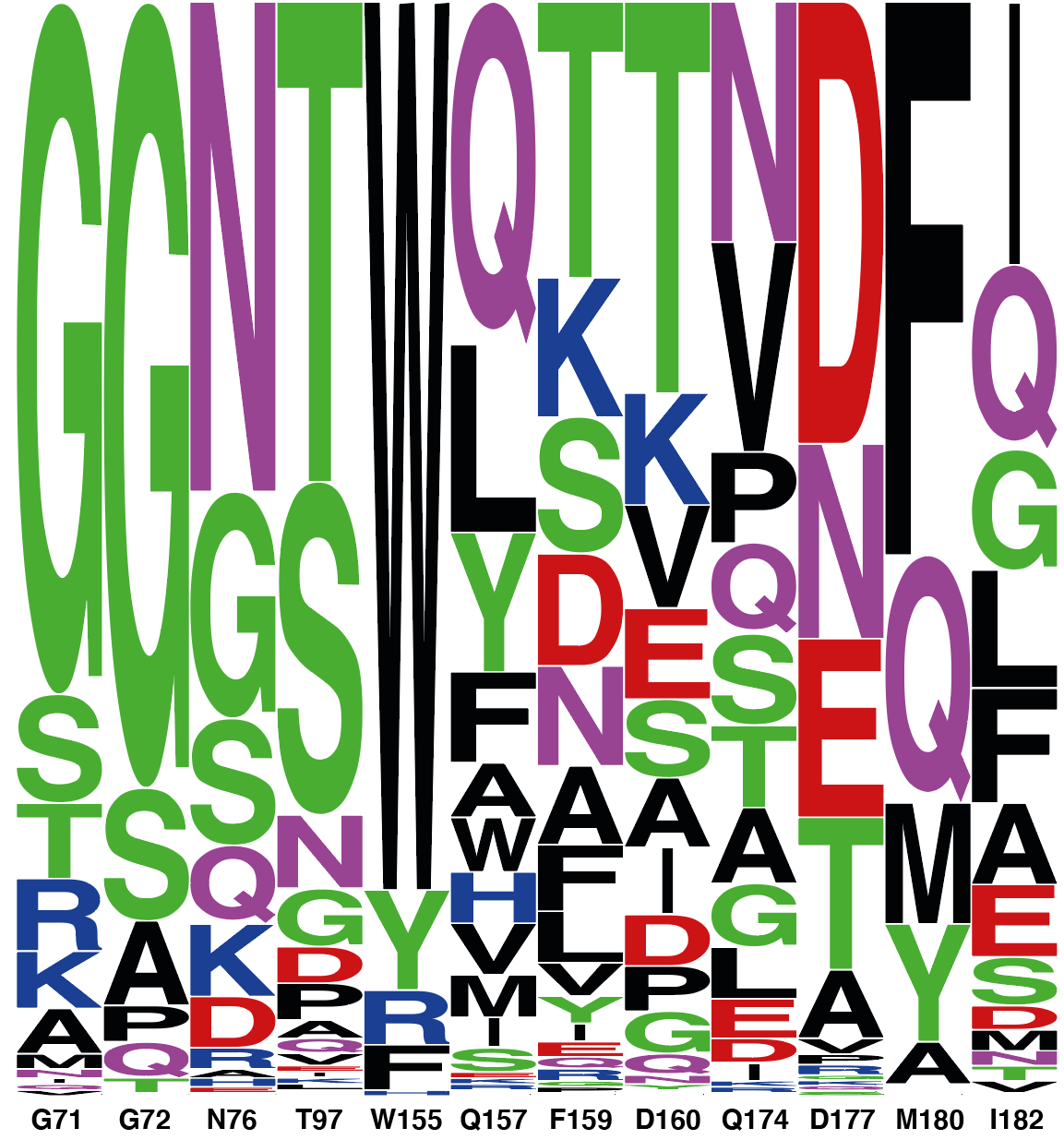


# **Figure S3. Analysis of amino acid conservation at selected positions.** The sequence logo frequency plot of the chosen positions was generated using WebLogo (<http://weblogo.berkeley.edu/>) based on a multiple sequence alignment (MSA) of 362 CDH sequences (see methods section for details).

# **Figure S4. Proof of concept for our interface engineering strategy on position D160.** (**A**) The pH profiles of *Ch*CDH WT (blue), D160G (red), D160R (green), and D160K (magenta) are shown as the pH-dependent IET rates (s^-1^). The pH profiles of the purified variants were measured in McIlvaine buffer in the presence of 30 mM lactose using the cyt *c* assay to determine the pH-dependent IET rates. (**B**) The IET rates at pH 7.5 are shown as arithmetic means ± 95 % confidence intervals of three independent experiments. Statistical analysis was performed by one-way ANOVA followed by Fisher’s Least Significant Difference. Asterisks indicate a statistical significance of p<0.0001 (***), p<0.001 (**), and p<0.05 (*) compared to *Ch*CDH WT.

# **Figure S5. Determination of cyt *c* assay threshold.** The IET rates based on five independent replicates measured with the cyt *c* assay is plotted against the relative standard deviation (RSD). As is apparent, RSD increases vastly at IET rates ≤0.01 s^-1^. Hence, the threshold for a reliable measurement of the IET rates of the engineered enzyme variants was set at ≤0.01 s^-1^ based on the limit of quantification for the cyt *c* assay. IET rates are shown as arithmetic means ± the 95% confidence interval.

# **Figure S6. pH Profiles of single-site variants.** The *Ch*CDH-WT is shown in black dots and variants are shown in white dots with black outline. The pH profiles were measured in McIlvaine buffer with 30 mM lactose as the substrate and 20 µM cyt *c* as the electron acceptor. Measurements were done in triplicates. Values are arithmetic means of three independent experiments. The pH profiles of the inactive variants G72K and I182K are not shown.

# **Figure S7. Correlation analysis of electron transfer rates and pH optimum.** IET rates at pH 4.5 (the WT pH optimum) and pH 7.5 of active variants are shown. The variants are colored according to their corresponding CYT interface region: IR-1 (green), IR-2 (purple), and IR-3 (orange). The outlier M180K is indicated by a black arrow. Analysis of the covariance between the IET rates at pH 4.5 and pH 7.5 showed a significant correlation. Pearson correlation coefficients were r=0.80 (95 % CI 0.33 to 0.95, p=0.0032) for all active variants and r=0.98 (95 % CI 0.89 to 0.99, p<0.0001) for all active variants excluding outlier M180K. The linear regression curve is shown in black with dotted lines indicating the 95% confidence interval and was modelled without the outlier M180K. Values are arithmetic means ± 95 % confidence intervals of three independent experiments. Inactive variants with IET rates of ≤0.01 s^-1^ (K72, G72K, N76K, W155K, and I182K) were not included in this analysis.

# **Figure S8. pH Profiles of multi-site variants.** The *Ch*CDH-WT is shown in black dots and variants are shown in white dots with black outline. The pH profiles of purified variants were measured in McIlvaine buffer in the presence of 30 mM lactose as a substrate and 20 µM cyt *c* as an electron acceptor. Measurements were done in triplicates. Values are arithmetic means of three independent experiments.

# **Figure S9. Cyclic voltammograms of WT *Ch*CDH and D160K.** (**A, B**) Non-catalytic CVs at increasing scan rates (10 to 100 mV s^-1^) and (**C, D**) overlay of non-catalytic (black solid line) and catalytic (in the presence of 5 mM lactose, red dashed line) CVs with the absolute catalytic current indicated by an arrow. Experimental conditions: 50 mM citrate buffer, pH 5.0, supplemented with 0.1 M KCl, 10 mV s^-1^ scan rate in (**A**). Shown is the second of three recorded cycles

# **Figure S10. Correlation of charge change of variants with current density in DET mode.** Current densities recorded during flow-injection analysis in DET mode at pH 5 in the presence of 5 mM lactose. Charge change is the sum of positively and negatively charged CYT interface residues that were changed as a result of inserting a mutant compared to the WT. Values are arithmetic means ± 95% confidence intervals of three independent experiments.
